# Supplementary material for: Barriers and opportunities in developing community-based maternal and child health surveillance: A mixed methods study in Depok, Indonesia
Source: PLoS One. 2025 Nov 17;20(11):e0332469. doi: 10.1371/journal.pone.0332469 (PMC12622817; doi:10.1371/journal.pone.0332469)
Supplement: S1 Table — (DOCX) [file pone.0332469.s001.docx]

**Supplemental Table 1. Characteristics of Respondents for the In-depth Interviews and Focus Group Discussions [Qualitative Analysis]**

|  |  | **In-depth Interviews**  **(n=23)** | **Focus Group Discussions**  **(n=2)** |
| --- | --- | --- | --- |
| Public Officials, City Level | n | 0 | 3 |
|  | Sex (%women) | - | 100 |
|  | Job Level (%senior) | - | 67 |
|  |  |  |  |
| Public Officials, District Level | n | 7^a^ | 1 |
|  | Sex, %women | 57 | 100 |
|  | Job Level, %senior | 71 | 100 |
|  |  |  |  |
| Public Officials, Subdistrict Level | n | 7^b^ | 2 |
|  | Sex, %women | 57 | 50 |
|  | Job Level, %senior | 29 | 100 |
|  |  |  |  |
| Medical Staff | n | 10^c^ | 3^e^ |
|  | Sex, %women | 100 | 100 |
|  | Job Level, %senior | 40 | 67 |
|  |  |  |  |
| Head of Neighbourhoods | n | 4^d^ | 0 |
|  | Sex, %women | 25 | - |
|  | >10 years of leadership, %yes | 25 | - |
|  |  |  |  |
| Health Volunteers | n | 3^d^ | 6^f^ |
|  | Sex, %women | 100 | 100 |
|  | >10 years of service, %yes | 67 | 67 |
|  |  |  |  |
| Community Members | n | 3^d^ | 0 |
|  | Sex, %women | 33 | - |
|  | >10 years of residency, %yes | 67 | - |

Data are presented as percentages (%) for categorical variables. ^a^Five staff from Sukmajaya, and two staff from Cimanggis Districts; ^b^Three staff from Curug, two from Tirtajaya, and two from Mekarjaya Subdistricts; ^c^Five staff from Cimanggis, and five from Sukmajaya Community Health Centers; ^d^At least one respondent from each of Curug, Tirtajaya, and Mekarjaya Subdistricts; ^e^Two GP coordinators from Cimanggis and Sukmajaya Community Health Centers, one paediatrician from Universitas Indonesia City Referral Hospital; ^f^Two health volunteers each from Curug, Tirtajaya, and Mekarjaya Subdistricts
