# Supplementary material for: Barriers and opportunities in developing community-based maternal and child health surveillance: A mixed methods study in Depok, Indonesia
Source: PLoS One. 2025 Nov 17;20(11):e0332469. doi: 10.1371/journal.pone.0332469 (PMC12622817; doi:10.1371/journal.pone.0332469)
Supplement: S2 Table — (DOCX) [file pone.0332469.s002.docx]

**Supplemental Table 2. Quantitative Analysis: Respondent’s Attitudes on Maternal and Child Health**

|  |  | **Men (n=301)** | | **Women (n=300)** | | **Total** | |
| --- | --- | --- | --- | --- | --- | --- | --- |
|  |  | **n** | **%** | **n** | **%** | **n** | **%** |
| Health monitoring for mothers and children is not obligatory to be implemented. | | | | | | | |
|  | Fully Agree | 2 | 0.6 | 3 | 1 | 5 | 0.8 |
|  | Agree | 14 | 4.8 | 21 | 7 | 35 | 5.9 |
|  | Disagree | 261 | 86.6 | 263 | 87.7 | 524 | 87.1 |
|  | Fully Disagree | 24 | 8.1 | 13 | 4.3 | 37 | 6.2 |
| Maternal blood tests will help check the health condition of pregnant mothers. | | | | | | | |
|  | Fully Agree | 19 | 6.2 | 17 | 5.7 | 36 | 6 |
|  | Agree | 276 | 91.5 | 282 | 94.1 | 557 | 92.8 |
|  | Disagree | 7 | 2.4 | 1 | 0.2 | 8 | 1.3 |
|  | Fully Disagree | 0 | 0 | 0 | 0 | 0 | 0 |
| Maternal blood tests will help in preparing blood donors if complications occur. | | | | | | | |
|  | Fully Agree | 23 | 7.5 | 19 | 6.2 | 41 | 6.9 |
|  | Agree | 275 | 91.3 | 280 | 93.6 | 556 | 92.4 |
|  | Disagree | 4 | 1.2 | 1 | 0.2 | 4 | 0.7 |
|  | Fully Disagree | 0 | 0 | 0 | 0 | 0 | 0 |
| Public health education related to maternal and child health is the obligation of health volunteers. | | | | | | | |
|  | Fully Agree | 15 | 5.1 | 8 | 2.8 | 24 | 4 |
|  | Agree | 215 | 71.5 | 222 | 74.3 | 438 | 72.9 |
|  | Disagree | 70 | 23.4 | 69 | 23 | 139 | 23.2 |
|  | Fully Disagree | 0 | 0 | 0 | 0 | 0 | 0 |
| Childbirth should be assisted by trained medical professionals such as midwives and doctors | | | | | | | |
|  | Fully Agree | 45 | 14.8 | 33 | 11.2 | 78 | 13 |
|  | Agree | 251 | 83.4 | 266 | 88.8 | 518 | 86.1 |
|  | Disagree | 5 | 1.7 | 0 | 0 | 5 | 0.9 |
|  | Fully Disagree | 0 | 0 | 0 | 0 | 0 | 0 |
| Pregnancy, childbirth, postpartum, and children's growth will go smoothly if local myths are followed. | | | | | | | |
|  | Fully Agree | 1 | 0.2 | 1 | 0.2 | 1 | 0.2 |
|  | Agree | 42 | 13.9 | 38 | 12.8 | 80 | 13.4 |
|  | Disagree | 244 | 81 | 251 | 83.8 | 495 | 82.4 |
|  | Fully Disagree | 15 | 4.9 | 9 | 3.2 | 24 | 4 |
| It is no problem if childbirth is assisted by a shaman/traditional healer. | | | | | | | |
|  | Fully Agree | 0 | 0 | 0 | 0 | 0 | 0 |
|  | Agree | 126 | 41.7 | 93 | 31 | 218 | 36.3 |
|  | Disagree | 164 | 54.4 | 196 | 65.5 | 360 | 59.9 |
|  | Fully Disagree | 12 | 3.9 | 11 | 3.6 | 23 | 3.8 |
