# Supplementary material for: Barriers and opportunities in developing community-based maternal and child health surveillance: A mixed methods study in Depok, Indonesia
Source: PLoS One. 2025 Nov 17;20(11):e0332469. doi: 10.1371/journal.pone.0332469 (PMC12622817; doi:10.1371/journal.pone.0332469)
Supplement: S3 Table — (DOCX) [file pone.0332469.s003.docx]

**Supplemental Table 3. Quantitative Analysis: Monitoring of Mothers' and Children's Health (n=601; 50% men)**

|  |  | **Pregnant Mothers** | | | **Delivering/Postpartum Mothers** | | | **Newborn Infants** | | |
| --- | --- | --- | --- | --- | --- | --- | --- | --- | --- | --- |
|  |  | **Men** | **Women** | **Total** | **Men** | **Women** | **Total** | **Men** | **Women** | **Total** |
|  |  | **%** | **%** | **%** | **%** | **%** | **%** | **%** | **%** | **%** |
| Would you allow the community to monitor the health of pregnant/delivering/postpartum mothers and newborn infants in your area? | | | | | | | | | | |
|  | Yes | 96.9 | 99 | 98 | 96.3 | 99.6 | 98 | 97.1 | 99.6 | 98,3 |
|  | No | 3.1 | 1 | 2 | 3.7 | 0.4 | 2 | 2.9 | 0.4 | 1,7 |
