# Supplementary material for: Barriers and opportunities in developing community-based maternal and child health surveillance: A mixed methods study in Depok, Indonesia
Source: PLoS One. 2025 Nov 17;20(11):e0332469. doi: 10.1371/journal.pone.0332469 (PMC12622817; doi:10.1371/journal.pone.0332469)
Supplement: S4 Table — (DOCX) [file pone.0332469.s004.docx]

**Supplemental Table 4. Quantitative Analysis: Perceptions Towards Healthcare Facilities (n=601; 50% men)**

|  |  | **Pregnant Mothers** | | | **Postpartum Mothers** | | | **Newborn Infants** | | |
| --- | --- | --- | --- | --- | --- | --- | --- | --- | --- | --- |
|  |  | **Men** | **Women** | **Total** | **Men** | **Women** | **Total** | **Men** | **Women** | **Total** |
|  |  | **%** | **%** | **%** | **%** | **%** | **%** | **%** | **%** | **%** |
| Do you find difficulties in reporting health cases related to pregnant mothers/postpartum mothers/newborn infants? | | | | | | | | | | |
|  | Yes | 0.8 | 1.2 | 1 | 1.6 | 1.2 | 1.4 | 1.4 | 1.4 | 1,4 |
|  | No | 79.7 | 86 | 82.8 | 78.3 | 86.2 | 82.2 | 78.1 | 85.4 | 81,7 |
|  | Not Sure | 19.6 | 12.8 | 16.2 | 20.1 | 12.6 | 16.4 | 20.6 | 13.2 | 16,9 |
|  |  |  |  |  |  |  |  |  |  |  |
| Do you find difficulties in obtaining healthcare services for pregnant mothers/postpartum mothers/newborn infants? | | | | | | | | | | |
|  | Yes | 1.5 | 1.6 | 1.6 | 1.5 | 1 | 1.3 | 1.4 | 1.4 | 1,4 |
|  | No | 79.5 | 86.4 | 82.9 | 79.1 | 87 | 83 | 78.8 | 86 | 82,4 |
|  | Not Sure | 19 | 12 | 15.5 | 19.4 | 12 | 15.7 | 19.8 | 12.6 | 16,2 |
