# Supplementary material for: Barriers and opportunities in developing community-based maternal and child health surveillance: A mixed methods study in Depok, Indonesia
Source: PLoS One. 2025 Nov 17;20(11):e0332469. doi: 10.1371/journal.pone.0332469 (PMC12622817; doi:10.1371/journal.pone.0332469)
Supplement: S5 Table — (DOCX) [file pone.0332469.s005.docx]

**Supplemental Table 5. Quantitative Analysis: Where to Report When Health Issues are Detected in Mothers and Children (n=601; 50% men)**

|  |  | **Pregnant Mothers** | | | **Delivering Mothers** | | | **Postpartum Mothers** | | | **Newborn Infants** | | |
| --- | --- | --- | --- | --- | --- | --- | --- | --- | --- | --- | --- | --- | --- |
|  |  | **Men** | **Women** | **Total** | **Men** | **Women** | **Total** | **Men** | **Women** | **Total** | **Men** | **Women** | **Total** |
|  |  | **%** | **%** | **%** | **%** | **%** | **%** | **%** | **%** | **%** | **%** | **%** | **%** |
| When there is an alarming health issue in pregnant mothers/delivering mothers/postpartum mothers/newborn infants, where should we report it? | | | | | | | | | | | | | |
|  | City’s Health Office | 0.6 | 0.2 | 0.4 | 0 | 0 | 0 | 0 | 0 | 0 | 0 | 0 | 0 |
|  | Hospital | 48 | 38.9 | 43.5 | 53.2 | 42.7 | 48 | 51.2 | 39.8 | 45.5 | 52.5 | 42.5 | 47.5 |
|  | Community Health Center | 47.8 | 42.5 | 45.2 | 45.4 | 40.8 | 43.1 | 46.1 | 39.9 | 43 | 48.5 | 41.4 | 45 |
|  | Clinic | 23.2 | 18.6 | 20.9 | 24.7 | 17 | 20.9 | 24.7 | 18.3 | 21.5 | 24.3 | 20.5 | 22.4 |
|  | Head of Village | 13.5 | 14.8 | 14.1 | 11.5 | 13.8 | 12.6 | 11.8 | 11.9 | 11.8 | 10.7 | 11.4 | 11 |
|  | Medical Professional | 9.7 | 16.1 | 12.9 | 10.4 | 13.5 | 12 | 9 | 15.6 | 12.3 | 9.3 | 13.9 | 11.6 |
|  | Public Official (District/Subdistrict) | 0 | 0 | 0 | 0 | 0 | 0 | 0 | 0 | 0 | 0 | 0.2 | 0.1 |
|  | Community Leader/Religious Leader | 0.2 | 0 | 0.1 | 0 | 0 | 0 | 0 | 0 | 0 | 0 | 0 | 0 |
|  | Health Volunteer | 13.3 | 12.7 | 13 | 11.9 | 11.3 | 11.6 | 11.3 | 9.3 | 10.3 | 11.1 | 9.3 | 10.2 |
|  | Family | 1.4 | 1.2 | 1.3 | 1.5 | 1.6 | 1.5 | 2.1 | 1.2 | 1.6 | 1.5 | 1 | 1.3 |
|  | Other | 2 | 3.5 | 2.7 | 1 | 2.6 | 1.8 | 0.8 | 3 | 1.9 | 0.8 | 2.8 | 1.8 |
|  | I do not report | 2.2 | 3.3 | 2.7 | 1.8 | 2.8 | 2.3 | 1.8 | 2.8 | 2.3 | 1.8 | 2.8 | 2.3 |
|  | I observe no cases | 4.4 | 3.3 | 3.9 | 4.6 | 3.7 | 4.2 | 5.2 | 5.6 | 5.4 | 5.2 | 5 | 5.1 |
|  | I don’t know if there are cases | 0.8 | 1.4 | 1.1 | 1.2 | 1.5 | 1.4 | 0.8 | 1.4 | 1.1 | 0.6 | 0.8 | 0.7 |
|  | I don’t know where to report | 2.3 | 1.6 | 1.9 | 2.3 | 0.4 | 1.4 | 2.5 | 0.6 | 1.6 | 2.5 | 0.4 | 1.5 |
|  |  |  |  |  |  |  |  |  |  |  |  |  |  |
| To your knowledge, are there feedback/responses from any relevant agencies regarding the report of health issues? | | | | | | | | | | | | | |
|  | Yes | 87.4 | 92.6 | 90 | 86.6 | 92.2 | 89.4 | 87 | 89.9 | 88.5 | 87.6 | 92 | 89.8 |
|  | No | 0 | 0 | 0 | 1.2 | 1 | 1.1 | 0.6 | 1 | 0.8 | 0 | 0.6 | 0.3 |
|  | I don’t know | 12.6 | 7.4 | 10 | 12.2 | 6.8 | 9.5 | 12.4 | 9.1 | 10.8 | 12.4 | 7.4 | 9.9 |
