# Supplementary material for: Barriers and opportunities in developing community-based maternal and child health surveillance: A mixed methods study in Depok, Indonesia
Source: PLoS One. 2025 Nov 17;20(11):e0332469. doi: 10.1371/journal.pone.0332469 (PMC12622817; doi:10.1371/journal.pone.0332469)
Supplement: Attachment — Submitted filename: PONE-D-24-59311-R1.pdf [file pone.0332469.s007.pdf]

PLOS ONE

# Barriers and Opportunities in Developing Community-Based Maternal and Child Health Surveillance: A Mixed Methods Study in Depok, Indonesia

--Manuscript Draft--

| | |
|---|---|
| **Manuscript Number:** | PONE-D-24-59311 |
| **Article Type:** | Research Article |
| **Full Title:** | Barriers and Opportunities in Developing Community-Based Maternal and Child Health Surveillance: A Mixed Methods Study in Depok, Indonesia |
| **Short Title:** | Barriers and Opportunities in Developing Community-Based Maternal and Child Health Surveillance in Indonesia |
| **Corresponding Author:** | Fathimah S Sigit, MD, PhD<br>Universitas Indonesia Faculty of Public Health: Universitas Indonesia Fakultas Kesehatan Masyarakat<br>Depok, Jawa Barat INDONESIA |
| **Keywords:** | Community-based surveillance; Maternal health, Child health; Health volunteers; pregnancy; Childbirth; stunting; Indonesia |
| **Abstract:** | Background<br>Comprehensive health surveillance for vulnerable populations, particularly mothers and children, is imperative beyond traditional survey methods to address issues occurring outside healthcare facilities and those linked with social stigma.<br><br>Methods<br>This embedded mixed-methods study aimed to identify factors contributing to an effective community-based surveillance system for maternal and child health. Quantitative data on knowledge, attitudes, perceptions, and stigmas were collected through an interviewer-assisted questionnaire in 300 households across three subdistricts in Depok. Qualitative insights into barriers and opportunities for detecting, reporting, and monitoring maternal and child health issues were obtained through in-depth interviews and focus group discussions with stakeholders, including public officials, community leaders, medical staff, and social workers.<br><br>Results<br>The household survey revealed that 22.5%, 24.1%, and 15.1% of respondents lacked knowledge of warning signs during pregnancy, childbirth, and newborn care, respectively. Men exhibited higher ignorance rates (34.2%, 35.2%, 23.2%) than women (10.7%, 12.9%, 7.0%). Most respondents (98%) supported community health monitoring. Thematic analysis unveiled barriers such as the high mobility of migrant families, inadequate capacity of health volunteers, stigma, delayed healthcare responses, and reluctance among families with middle-high socioeconomic status. Contrariwise, opportunities included positive community perceptions of surveillance, ongoing community empowerment activities, active roles of health volunteers, potential stakeholder collaboration, and digital communication channels.<br><br>Discussion<br>Public health education primarily targeting men is indispensable to enhancing their awareness of maternal and child health issues. Addressing identified barriers and leveraging opportunities could establish a sustainable and well-received community-based surveillance system, crucial for ensuring the health of mothers and children. |
| **Order of Authors:** | Fathimah S Sigit, MD, PhD |
| | Fitra Yelda, M.Epid |
| | Dumilah Ayuningtyas, Professor |
| | Asri C Adisasmita, Professor |
| | Sabarinah Prasetyo, Professor |
| **Additional Information:** | |

Powered by Editorial Manager® and ProduXion Manager® from Aries Systems Corporation

| Question | Response |
| --- | --- |
| **Financial Disclosure**<br><br>Enter a financial disclosure statement that describes the sources of funding for the work included in this submission. Review the submission guidelines for detailed requirements. View published research articles from *PLOS ONE* for specific examples.<br><br>This statement is required for submission and **will appear in the published article** if the submission is accepted. Please make sure it is accurate.<br><br>**Funded studies**<br>Enter a statement with the following details:<br>• Initials of the authors who received each award<br>• Grant numbers awarded to each author<br>• The full name of each funder<br>• URL of each funder website<br>• Did the sponsors or funders play any role in the study design, data collection and analysis, decision to publish, or preparation of the manuscript?<br><br>Did you receive funding for this work? | Yes |
| Please add funding details.<br>as follow-up to "**Financial Disclosure**<br><br>Enter a financial disclosure statement that describes the sources of funding for the work included in this submission. Review the submission guidelines for detailed requirements. View published research articles from *PLOS ONE* for specific examples.<br><br>This statement is required for submission and **will appear in the published article** if the submission is accepted. Please make sure it is accurate. | This study is supported by the Indonesia Endowment Fund for Education (Indonesian: Lembaga Pengelola Dana Pendidikan; LPDP), Grant Number: NKB-696/UN2.RST/HKP.05.00/2021 PRIME (Partnership in Research Indonesia and Melbourne).The funder had no role in study design, data collection and analysis, decision to publish, or preparation of this manuscript. |

Powered by Editorial Manager® and ProduXion Manager® from Aries Systems Corporation

| | |
|---|---|
| **Funded studies**<br>Enter a statement with the following details:<br>• Initials of the authors who received each award<br>• Grant numbers awarded to each author<br>• The full name of each funder<br>• URL of each funder website<br>• Did the sponsors or funders play any role in the study design, data collection and analysis, decision to publish, or preparation of the manuscript?<br><br>Did you receive funding for this work?" | |
| Please select the country of your main research funder (please select carefully as in some cases this is used in fee calculation).<br>as follow-up to "**Financial Disclosure**<br><br>Enter a financial disclosure statement that describes the sources of funding for the work included in this submission. Review the submission guidelines for detailed requirements. View published research articles from *PLOS ONE* for specific examples.<br><br>This statement is required for submission and **will appear in the published article** if the submission is accepted. Please make sure it is accurate.<br><br>**Funded studies**<br>Enter a statement with the following details:<br>• Initials of the authors who received each award<br>• Grant numbers awarded to each author<br>• The full name of each funder<br>• URL of each funder website<br>• Did the sponsors or funders play any role in the study design, data collection and analysis, decision to publish, or preparation of the manuscript?<br><br>Did you receive funding for this work?" | INDONESIA - ID |
| **Competing Interests** | The authors have declared that no competing interests exist. |

| | |
|---|---|
| Use the instructions below to enter a competing interest statement for this submission. On behalf of all authors, disclose any competing interests that could be perceived to bias this work—acknowledging all financial support and any other relevant financial or non-financial competing interests.<br><br>This statement is required for submission and **will appear in the published article** if the submission is accepted. Please make sure it is accurate and that any funding sources listed in your Funding Information later in the submission form are also declared in your Financial Disclosure statement.<br><br>View published research articles from *PLOS ONE* for specific examples.<br><br>**NO authors have competing interests**<br><br>Enter: *The authors have declared that no competing interests exist.*<br><br>**Authors with competing interests**<br><br>Enter competing interest details beginning with this statement:<br><br>*I have read the journal's policy and the authors of this manuscript have the following competing interests: [insert competing interests here]*<br><br>* typeset | |
| **Ethics Statement**<br><br>Enter an ethics statement for this submission. This statement is required if the study involved:<br><br>• Human participants<br>• Human specimens or tissue<br>• Vertebrate animals or cephalopods<br>• Vertebrate embryos or tissues | The Research and Community Engagement Ethical Committee, Faculty of Public Health, Universitas Indonesia, had approved the ethics of this study (No: Ket-564/UN2.F10.D11/PPM.00.02/2022). |

Powered by Editorial Manager® and ProduXion Manager® from Aries Systems Corporation

| | |
|---|---|
| • Field research<br><br>Write "N/A" if the submission does not require an ethics statement.<br><br>General guidance is provided below. Consult the submission guidelines for detailed instructions. **Make sure that all information entered here is included in the Methods section of the manuscript.** | |

| | |
|---|---|
| **Format for specific study types**<br><br>**Human Subject Research (involving human participants and/or tissue)**<br>• Give the name of the institutional review board or ethics committee that approved the study<br>• Include the approval number and/or a statement indicating approval of this research<br>• Indicate the form of consent obtained (written/oral) or the reason that consent was not obtained (e.g. the data were analyzed anonymously)<br><br>**Animal Research (involving vertebrate animals, embryos or tissues)**<br>• Provide the name of the Institutional Animal Care and Use Committee (IACUC) or other relevant ethics board that reviewed the study protocol, and indicate whether they approved this research or granted a formal waiver of ethical approval<br>• Include an approval number if one was obtained<br>• If the study involved *non-human primates*, add *additional details* about animal welfare and steps taken to ameliorate suffering<br>• If anesthesia, euthanasia, or any kind of animal sacrifice is part of the study, include briefly which substances and/or methods were applied<br><br>**Field Research**<br><br>Include the following details if this study involves the collection of plant, animal, or other materials from a natural setting:<br>• Field permit number<br>• Name of the institution or relevant body that granted permission | |
| **Data Availability**<br><br>Authors are required to make all data underlying the findings described fully available, without restriction, and from the time of publication. PLOS allows rare exceptions to address legal and ethical concerns. See the PLOS Data Policy and FAQ for detailed information. | No - some restrictions will apply |

| | |
|---|---|
| A Data Availability Statement describing where the data can be found is required at submission. Your answers to this question constitute the Data Availability Statement and **will be published in the article**, if accepted.<br><br>**Important:** Stating 'data available on request from the author' is not sufficient. If your data are only available upon request, select 'No' for the first question and explain your exceptional situation in the text box.<br><br>Do the authors confirm that all data underlying the findings described in their manuscript are fully available without restriction? | |
| **Describe where the data may be found in full sentences. If you are copying our sample text, replace any instances of XXX with the appropriate details.**<br><br>• If the data are **held or will be held in a public repository**, include URLs, accession numbers or DOIs. If this information will only be available after acceptance, indicate this by ticking the box below. For example: *All XXX files are available from the XXX database (accession number(s) XXX, XXX.).*<br>• If the data are all contained **within the manuscript and/or Supporting Information files**, enter the following: *All relevant data are within the manuscript and its Supporting Information files.*<br>• If neither of these applies but you are able to provide **details of access elsewhere**, with or without limitations, please do so. For example:<br><br>*Data cannot be shared publicly because of [XXX]. Data are available from the XXX Institutional Data Access / Ethics Committee (contact via XXX) for researchers who meet the criteria for access to confidential data.*<br><br>*The data underlying the results presented in the study are available from (include the name of the third party* | Data cannot be shared publicly because of confidentiality issues, as there was some personal information related to the participants. Data are available from the Research and Community Engagement Ethical Committee of the Faculty of Public Health Universitas Indonesia (email: fkmui@ui.ac.id), for researchers who meet the criteria for access to the confidential data. |

Powered by Editorial Manager® and ProduXion Manager® from Aries Systems Corporation

| | |
|---|---|
| *and contact information or URL).*<br>• This text is appropriate if the data are owned by a third party and authors do not have permission to share the data.<br><br>* typeset | |
| Additional data availability information: | |

Powered by Editorial Manager® and ProduXion Manager® from Aries Systems Corporation

Manuscript

Click here to access/download;Manuscript;PRIME Study Manuscript 1 - FINAL.docx

# Barriers and Opportunities in Developing Community-Based Maternal and Child Health Surveillance: A Mixed Methods Study in Depok, Indonesia

**Fathimah S. Sigit[1*]**, Fitra Yelda[2], Dumilah Ayuningtyas[3], Asri C. Adisasmita[4], Sabarinah Prasetyo[5]

[1]Department of Public Health Nutrition, Faculty of Public Health, Universitas Indonesia

[2]Center for Health Research, Faculty of Public Health, Universitas Indonesia

[3]Department of Health Policy and Administration, Faculty of Public Health, Universitas Indonesia

[4]Department of Epidemiology, Faculty of Public Health, Universitas Indonesia

[5]Department of Biostatistics and Population Studies, Faculty of Public Health, Universitas Indonesia

**Corresponding Author*. *Correspond to: fathimah10@ui.ac.id*

**Abstract**

**Background**

Comprehensive health surveillance for vulnerable populations, particularly mothers and children, is imperative beyond traditional survey methods to address issues occurring outside healthcare facilities and those linked with social stigma.

**Methods**

This embedded mixed-methods study aimed to identify factors contributing to an effective community-based surveillance system for maternal and child health. Quantitative data on knowledge, attitudes, perceptions, and stigmas were collected through an interviewer-assisted questionnaire in 300 households across three subdistricts in Depok. Qualitative insights into barriers and opportunities for detecting, reporting, and monitoring maternal and child health issues were obtained through in-depth interviews and focus group discussions with stakeholders, including public officials, community leaders, medical staff, and social workers.

**Results**

The household survey revealed that 22.5%, 24.1%, and 15.1% of respondents lacked knowledge of warning signs during pregnancy, childbirth, and newborn care, respectively. Men exhibited higher ignorance rates (34.2%, 35.2%, 23.2%) than women (10.7%, 12.9%, 7.0%). Most respondents (98%) supported community health monitoring. Thematic analysis unveiled barriers such as the high mobility of migrant families, inadequate capacity of health volunteers, stigma, delayed healthcare responses, and reluctance among families with middle-high socioeconomic status. Contrariwise, opportunities included positive community perceptions of surveillance, ongoing community empowerment activities, active roles of health volunteers, potential stakeholder collaboration, and digital communication channels.

**Discussion**

Public health education primarily targeting men is indispensable to enhancing their awareness of maternal and child health issues. Addressing identified barriers and leveraging opportunities could establish a sustainable and well-received community-based surveillance system, crucial for ensuring the health of mothers and children.

## Introduction

Public health surveillance is the systematic collection, collation, and analysis of data for public health purposes. It is an integral part of creating and maintaining healthcare services, as it helps stakeholders detect health problems occurring in society. Information from surveillance may be used to help decision-makers identify modifiable factors in health issues for continued healthcare improvement, or plan actionable public health responses (1–3).

Besides conventional public health surveillance that requires many resources to be conducted, the COVID-19 pandemic has shown us the importance of community-based surveillance, particularly in low- and middle-income countries with limited resources. Community-based surveillance (CBS) is a systematic detection and reporting of events of public health significance within a community by community members, that may serve as an early warning of certain public health issues, which can lead to immediate decision-making of preventive or curative measures (2). During the COVID-19 pandemic, CBS was used widely worldwide, as community members provided prompt reporting of a probable case of the disease in their community to healthcare providers via a 'real-time' communication network. In the context of COVID-19, CBS helps interrupt transmission through early detection and follow-up response from health authorities of a potential positive case in the community (4). Unlike the conventional approach in which healthcare facilities usually being the ones gathering data for surveillance, in CBS the community members can report an incident of a (suspected) case in their community, and relay the findings to the integrated reporting and record systems. Hence, it empowers the community to participate in managing public health issues and improving disease preparedness (5,6).

Among public health issues in Indonesia, maternal and child health problems remain one of the leading national public health concerns. Despite steadily decreasing, maternal deaths and other related complications of pregnancy and childbirth are still occurring in Indonesia. For example, chronic energy deficiency is still commonly found in pregnant women, particularly those living in rural areas. Poor pregnancy outcomes such as low birth weight or premature-born babies are also still found. Previous studies have shown that worse pregnancy outcomes more commonly occur in countries with limited health registration systems, as the lack of data hinders planning and monitoring the progress of public health policies. Thus, in these low-resource settings, community-based surveillance may become an alternative registration system to provide those data (7–9).

Although Indonesia already has an integrated health information and reporting system that was collected by healthcare facilities across the nation, this system is not fully well-established. For example, in rural areas with limited facilities, reports are still commonly collected paper-based, which

led to the data not providing a real-time picture of health issues in the population, as it takes time to aggregate the information (1). This system was also not representative of the underprivileged population who did not access, or had no access to, the formal healthcare facilities, which makes cases (e.g., maternal deaths) that occurred outside healthcare facilities unreported (10). These gaps may be addressed by community-based surveillance, as a complementary or integral part of the conventional information system (1,9,10).

Nevertheless, despite its benefits and low related costs, CBS is currently not much implemented in Indonesia. Therefore, to help set the foundation for an effective, efficient, and well-received community-based surveillance system, particularly for maternal and child health issues, this study aimed to identify barriers and opportunities related to the surveillance in three sub-districts in Depok, Indonesia.

## Methods

*Study Design and Population*

The target population in this study were residents living in the city of Depok, Indonesia. Being a supporting neighbour city of the capital Jakarta, Depok is an urban city demographically characterised by its many migrant residents coming from rural areas across Indonesia. Similar to other areas in Indonesia, the primary healthcare service for the population is provided by the Community Health Center, a free-for-public, government-funded healthcare facility.

This study has a mixed methods approach as it employs both quantitative and qualitative data collection and analysis methods. Using an embedded design, the qualitative study provided data supporting the quantitative study. Study recruitment and data collection were performed between 1 November 2022 and 1 October 2023.

*Quantitative Approach: The Interviewer-assisted Questionnaire*

Quantitative data were collected from 300 households residing in three subdistricts in Depok (Mekarjaya, Curug, and Tirtajaya) with an interviewer-assisted questionnaire. The three subdistricts were chosen by the Depok City Health Office, based on their relatively higher prevalence of maternal and children health issues compared to other subdistricts. A combined cluster and systematic random sampling method was used to select the households. Initially, twenty neighbourhoods (Indonesian: ‘Rukun Tetangga’; ‘RT’) from the three subdistricts were chosen as the clusters with the probability proportional to size method. Then, five households per neighbourhood were selected randomly. Data

enumerators visited the households and asked the heads of the households and their spouses to complete the questionnaires.

The cross-sectional questionnaire aimed to assess the level of knowledge, attitudes, and stigmas related to maternal and neonatal health issues and surveillance. These variables were evaluated as understanding the cultural-social norms, perceptions, and attitudes towards health issues is essential to designing a well-accepted relevant surveillance system (8).

*Qualitative Approach: In-depth interviews and Focus Group Discussions*

To gain comprehensive qualitative insights from multiple perspectives of the community, informants for the in-depth interviews were selected purposively, including community leaders, health volunteers, medical staff at the Community Health Centre, and stakeholders from the local administrative and health authorities at the subdistrict, district, and city levels. The interviews were done during daytime, working hours, at their offices or places as visited by the data enumerators.

After the interviews, focus group discussions (FGDs) with representatives of the stakeholders and elements of the community were conducted to identify potential solutions or best practices in the community that are applicable to developing a community-based surveillance system. During the FGDs, the moderator guided the discussion using guide topics. The FGDs were held at the University of Indonesia, Faculty of Public Health, and were fully recorded and transcribed by study assistants.

For interviews and FGDs, there was no time limitation per data collection session, but they ended when data saturation was reached, or when no additional information was obtained. The interviews and FGDs were performed in Indonesian language. The involvement of multiple stakeholders/policymakers was aimed to help in planning actionable interventions, as well as reinforcing local policies.

*Data Analysis*

For the quantitative data, a weighted analysis was performed to account for the sampling design. Data were presented as mean (SD) for continuous variables or proportion (%) for categorical variables. For the qualitative data, all interviews and FGDs sessions were transcribed and re-read, and common themes were identified using the framework approach and qualitative data analysis software (ATLAS.ti Scientific Software Development GmbH). After familiarisation with the data, the main themes were highlighted, and thematic analysis was performed.

*Ethics Statement*

This study had been reviewed and approved by the Ethics Committee of the Faculty of Public Health, Universitas Indonesia (No.Ket-563/UN2.F10.D11/PPM.00.02/2022). All participants signed written

informed consent. No minors were included, and no personal information that could identify individual participants was reported in the present study.

## Results

*Quantitative Analysis: Descriptive Characteristics of the Study Population* ***[Table 1]***

The quantitative study population has an equal proportion of men (50.1%) and women (49.9%). Most respondents who filled in the questionnaires were the heads of the households (43.5%) or their spouses (43.9%). The majority of respondents (87.7%) were married, and approximately half of them (49.9%) were high school graduates. Two-thirds (69.1%) of the respondents never smoked, whereas 24.8% are current smokers.

*Knowledge of Warning Signs in Pregnancy, Childbirth, and Newborn Babies* ***[Table 2]***

When asked about the warning signs that have to be looked into in pregnant mothers, bleeding/haemorrhage (40.5%) and hypertension (18.6%) were the most chosen options from the answers. A quarter (22.5%) of participants responded that they did not know the warning signs. The proportion of respondents who answered that they did not know the warning signs was disproportionately higher in men (34.2%) than women (10.7%).

In regard to warning signs during childbirth, around 25.1% of respondents chose premature rupture of the membrane, 42.7% answered massive bleeding during and after delivery, and 10.9% answered prolonged contraction or prolonged labour. A quarter respondents (24.1%) answered that they did not know the warning signs during childbirth, with again a disproportionate proportion between men (35.2%) and women (12.9%). When asked about warning signs in newborn babies, the most chosen answers are jaundice (54%), babies not crying (19.3%), and small born or low birth weight babies (17.6%). Around 15.1% answered that they did not know the warning signs in newborn babies, and this is more so in male (23.2%) than female (7.0%) respondents.

*Attitudes on Maternal and Child Health Issues*

The majority of respondents (87.1%) perceive that health monitoring is essential and should be made compulsory. Around 72.9% of respondents agreed that counselling to maintain mothers' and children's health is a duty of health volunteers, and 86.1% agreed that childbirth should be assisted by trained medical professionals, such as midwives and doctors. The majority (82.4%) disagree that pregnancy, delivery, and child growth can happen well if local myths are followed, and 59.9% disagree if childbirth is assisted by non-medical professionals (e.g., shamans). When asked about their approval of pregnant

mothers, births, and newborn babies in their community being monitored, the majority of (98%) respondents said that they would approve such monitoring to be conducted **[Supplemental Table 1-2]**.

*Perceptions Towards Case Reporting and Healthcare Facilities*

Approximately 82% of respondents did not have difficulties reporting health issues related to pregnant mothers, childbirth, or sick newborns. The majority (>82%) also did not have problems obtaining healthcare services. If issues related to pregnant mothers, childbirth, postpartum mothers, and newborn babies occur in the community, respondents believe that they should report to the Community Health Center (43.0-45.2%), nearby hospital (43.5-48.0%), clinics (20.9-22.4%), social worker (10.2-13.0%), health professionals (11.6-12.9%), and community leaders (11.0%-14.1%) **[Supplemental Table 3-4]**.

*<u>Qualitative Analysis: Barriers to Establishing a Community-Based Surveillance System for Maternal and Child Health</u>* ***<u>[Table 3]</u>***

1. *The High Mobility of Residents*

The biggest identified challenge is the high mobility of incoming and outgoing migrants from rural areas all over the country who reside temporarily in Depok to seek jobs in the city or the capital, Jakarta. These migrants bring along their families, including their children, who often have undernutrition problems (stunting or wasting). This particular theme emerges repeatedly and consistently from many stakeholders, such as public officials at the district and subdistrict levels, medical staff from the Community Health Centre, and local health volunteers. Despite some intervention programs having been carried out to prevent stunting, as the influx exceeds the outflow of migrant families, the number of children with stunting always appears to be high in the city.

2. *Inadequate Capacity of the Health Volunteers*

Another challenge was the inadequate capacity of the health volunteers, which hindered the delivery of surveillance. For example, as health volunteers are generally older, many are not digitally adept at utilising online reporting tools. Also, it was observed that the health volunteers often did not perform anthropometric measurements (weight and height) in a correct manner to the children at the local 'Integrated Health Service Post' (Indonesian: Posyandu), which is an activity organised regularly by health volunteers in the community to monitor the health of pregnant mothers and children,

supervised by the village midwives. This measurement error led to inaccurate reporting of the children's nutritional status.

*3. Stigma Related to Sensitive Maternal and Child Health Issues*

Sometimes families or community members avoid surveillance or house visits by health volunteers, especially in cases the public sees as shame, such as when mothers are pregnant out of marriage. The avoidance was also shown by their hesitancy to have their conditions checked or examined by health professionals.

*4. Late Response from Healthcare Providers*

Another potential barrier was the sometimes late response from healthcare providers after a case was reported. This sometimes makes community members less eager to report when other new issues are found in their community, as they thought their report was meaningless.

*5. Unwillingness to Participate in Families with Middle-High Socioeconomic Status*

In addition, sometimes families reject visits from health volunteers simply because they do not know the enumerator/visitor or have no time to be surveyed. This was particularly often in higher-income neighbourhood clusters with middle-high socioeconomic status families.

*Qualitative Analysis: Opportunities in Establishing the Community-Based Surveillance* ***[Table 3]***

*1. The Community's Positive Perceptions on Surveillance*

Most residents perceived that detecting, recording, and reporting certain medical conditions were necessary for health monitoring. There was, in fact, already a reporting mechanism from the community to the Community Health Centre. This was done mainly by health volunteers conducting house visits to the community members who had a suspected case, or the community members informed the health volunteers when they found a suspected case, and the health volunteers relayed the information to the Community Health Centre. The medical staff at the Community Health Centre then verify the instances by checking the health conditions of the individuals with the suspected case.

*2. The Existing Community Empowerment Activities*

A best practice that has been organised routinely by community members to their community is the 'Blessed Friday' program, in which in this program, nutritious food products (raw or ready-to-eat) are distributed to families in need every Friday of the week. These foods are prepared by the community for families with socioeconomic disadvantages, and those with stunted children are given priority as

targets for this program. Besides the 'Blessed Friday' program, another best practice is the weekly Qurán recitation events, in which in this mass congregation, the community/religious leaders often remind the public to check their condition at the Community Health Center and help those who are ill.

*3. The Active Role of Health Volunteers*

Health volunteers play a major role in identifying health issues in the community. One of their primary roles is to organise the routine Integrated Health Service Post activities (‘Posyandu’), during which the general health of pregnant mothers and children’s growth is monitored. The volunteers then report if any high-risk pregnancies are detected, as well as the anthropometric measurement data of the children, to the Community Health Center. In cases of high-risk pregnancies, the volunteers also routinely visit the mothers at home to monitor their general health status.

*4. Potential Collaboration between Stakeholders and Community Organisations*

Monthly coordination meetings between health volunteers, district and subdistrict officials, and medical staff of the community health centre are regularly held to evaluate and solve health issues in the community. However, they believe that more stakeholders should be invited or involved in the coordination meetings, such as private healthcare services, academic institutions, local (para)military force, youth organisations, and the office of communication and informatics, as they have distinguished roles that each contribute to better surveillance system.

For example, involving private hospitals and midwife practices in the area may strengthen the referral network between healthcare facilities. Academic institutions may aid in providing health information sessions regarding a particular issue in the community, or help detect health issues and suggest relevant potential interventions by doing research. The local (para)military officers may be involved in surveillance activities (e.g., household visits), as the community tend to consider personnel ‘in uniform’ more seriously. Youth organisations can be asked for their ideas as they are generally more well-educated and innovative, and may explore new funding opportunities to finance the community-based surveillance system. Meanwhile, the Information Office (Diskominfo) can help in conveying public health messages or reducing stigmas of certain sensitive issues.

*5. Digital Communication Channels*

Developing a user-friendly digital tool to record cases is potentially welcomed by the community, and maintenance should be prepared. In fact, in the subdistrict of Tirtajaya, there is already an online Google sheet that can be filled by households and health volunteers whose data is sent directly to the Community Health Centre. This online spreadsheet is called the ‘Healthy Family Card’, in which households independently report their health issues to the tool.

Besides this online spreadsheet, digital mobile applications, such as WhatsApp groups, are also seen as efficient coordination channels between stakeholders (community health centre staff and the district and subdistrict officials), as well as information source for the community during the Covid-19 pandemic. For example, there is a specific WhatsApp group dedicated to pregnant mothers in the community, in which in the group the health volunteers routinely provide information on maintaining a healthy pregnancy. Social media such as Instagram and TikTok are also seen as communication channels that are particularly relevant and preferred by the younger generation of mothers.

## Discussion

In this study, we aimed to identify barriers and opportunities that may contribute to the development of a community-based surveillance system for maternal and child health. We used a quantitative approach to assess the knowledge, attitudes, and perceptions of the residents regarding surveillance and maternal and child health issues, in combination with a qualitative approach by interviewing and conducting focus group discussions with multiple stakeholders in the community.

We observed that around 15.1%-24.1% of the general public appeared to have insufficient knowledge of maternal and child health issues, and this is disproportionately higher in men (23.2%-35.2%) than women (7.0%-12.9%). Most respondents (98%) approve the monitoring of pregnant mothers, childbirth, and newborn babies, and local stakeholders perceived community-based surveillance as an important method to collect complementary data on health events, alongside the conventional surveillance system.

Several opportunities and barriers were observed in relation to the implementation of community-based surveillance. The barriers are the high mobility of incoming and outgoing migrant workers with their children, the lack of capacity of the health volunteers, stigma related to sensitive maternal and child health issues, late response from healthcare providers, and unwillingness to participate in families with middle-high socioeconomic status. On the other hand, the opportunities are the community's positive perceptions of surveillance, the existing community empowerment activities, the active role of health volunteers, potential collaboration between stakeholders and community organisations, and digital communication channels.

From our quantitative analysis, we observed that a substantial proportion of adults in the study did not have adequate knowledge of warning signs of maternal and child health issues, particularly regarding pregnancy, childbirth, and newborn care. This problem is a lot worse in men than women, and is potentially detrimental as it may lead to less involvement of husbands as key decision-makers

in the household, which then influences whether the mothers and the newborns can obtain required healthcare services during critical periods (11,12). Thus, public health education to raise awareness on maternal and child health issues should also prioritise men as the target.

Comparing our qualitative findings to previous studies, our observation that the high mobilization of residents is a barrier to employing community-based surveillance is aligned with previous studies. Incoming migrant families living temporarily in rented houses are often unregistered in the villages and thus cannot be reached by health volunteers. Special attention has to be given to this specific group of society, as studies in other countries found that the risk of stunting increases as the rate of urbanization rises. Possible explanations are financial strains, lack of iron-rich diets, and maternal anaemia (13), as the ‘urban poors’ are less likely to access healthcare services (14). In addition, as they are likely not registered in any health systems, these populations might not have any health insurance, including subsidized healthcare insurance from the government. Thus, extra community effort should be encouraged to "catch" this population segment, for example, by community members relaying the information about incoming migrant families in their neighbourhood to the local head of village. When these unregistered migrant families are recorded in the health system, better accessibility to healthcare will follow.

Our finding was also coherent with previous studies, which observed that stigma is a barrier to accessing public healthcare services (15,16), leading to decreased quality and continuity of care (17). Nevertheless, our finding that households with high socioeconomic status, or those who live in executive neighbourhood complexes, were less likely to participate in community-based surveillance was quite the contrary from what was observed in Western countries, as they found that those with high socioeconomic status and education were the ones having high response-rate of both web surveys and in-person surveys (18–20). This finding underscores the specific phenomenon occurring in Indonesia, highlighting the need for a better approach for the wealthy to increase their participation in community surveys.

Another common theme that emerges as a prominent opportunity is the potential use of digital communication channels as an essential tool in community-based surveillance. This is aligned with a previous study which observed that digital applications or electronic-based reporting systems contributed to the well-acceptability of surveillance in the community. In this prior study, they observed that mobile phones were perceived by society as an accessible and practical medium that can relay information regarding health events quickly, and seen by the community to be linked to better healthcare services (21). Incorporating mobile phones as a tool for data reporting by community

members may also provide real-time data with low utilisation costs. This also promotes a timely response from health authorities if a health issue is detected (22).

A particular theme that emerges as both an opportunity and a challenge is the role of health volunteers, or social health workers. It is well established that health volunteers have pivotal roles in the community, and this was clearly seen during the COVID pandemic when there was a massive shortage of trained medical professionals. Their existence is crucial in promoting and delivering public health interventions and surveillance programs, and their contributions to the community are done without costly means and reach the population widely, including rural and remote areas (23,24). In implementing community-based surveillance, the health volunteers act as informants who have a central role in tracking and collecting information on health events, for example through house visits (10). Nevertheless, we observed that systematic errors in their work might result in misidentifications of health issues, which is aligned with a previous study that found that lack of capacity was identified as a factor that hindered the effective delivery of health volunteer roles. Thus, continuous capacity building should be advocated for the sustainability of health volunteers (25–27). Also, although most volunteers understood the budget constraint and were not demanding remuneration, they desired that their work be recognized and appreciated by means other than money (28,29). Thus, proper rewards for their services or non-financial incentives should also be considered (25,30).

Finally, to summarize and conclude this study, we observed that there is still a considerable proportion of the population who have inadequate knowledge of maternal and child health issues, and this is more particularly so in men than women, implying that health education should also include men to encourage their involvement in ensuring the health of mothers and children. Community-based surveillance is potentially well-received, and mitigating barriers and employing opportunities observed in this study may help establish a sustainable community-based surveillance system.

**References**


1. Pasha O, Saleem S, Ali S, Goudar SS, Garces A, Esamai F, et al. Maternal and newborn outcomes in Pakistan compared to other low and middle income countries in the Global Network's Maternal Newborn Health Registry: an active, community-based, pregnancy surveillance mechanism. Reprod Health. 2015;12 Suppl 2(Suppl 2):S15.

2. Wight D, Wimbush E, Jepson R, Doi L. Six steps in quality intervention development (6SQuID). J Epidemiol Community Health. 2016 May;70(5):520–5.

3. Technical Contributors to the June 2018 WHO meeting. A definition for community-based surveillance and a way forward: results of the WHO global technical meeting, France, 26 to 28 June 2018 [Internet]. Euro Surveillance; 2019 [cited 2023 Oct 18]. Available from: https://doi.org/10.2807/1560-7917.ES.2019.24.2.1800681

4. Brookes VJ, Kennedy E, Dhagapan P, Ward MP. Qualitative Research to Design Sustainable Community-Based Surveillance for Rabies in Northern Australia and Papua New Guinea. Front Vet Sci. 2017;4:19.

5. Guerra J, Acharya P, Barnadas C. Community-based surveillance: A scoping review. PLoS One. 2019;14(4):e0215278.

6. McNabb SJN, Chungong S, Ryan M, Wuhib T, Nsubuga P, Alemu W, et al. Conceptual framework of public health surveillance and action and its application in health sector reform. BMC Public Health. 2002;2:2.

7. McClure EM, Garces AL, Hibberd PL, Moore JL, Goudar SS, Saleem S, et al. The Global Network Maternal Newborn Health Registry: a multi-country, community-based registry of pregnancy outcomes. Reprod Health. 2020 Nov 30;17(Suppl 2):184.

8. Shannon C, Hurt C, Soremekun S, Edmond K, Newton S, Amenga-Etego S, et al. Implementing effective community-based surveillance in research studies of maternal, newborn and infant outcomes in low resource settings. Emerg Themes Epidemiol. 2022 Jan 12;19(1):1.

9. Adomako J, Asare GQ, Ofosu A, Iott BE, Anthony T, Momoh AS, et al. Community-based surveillance of maternal deaths in rural Ghana. Bull World Health Organ. 2016 Feb 1;94(2):86–91.

10. Basera TJ, Schmitz K, Price J, Willcox M, Bosire EN, Ajuwon A, et al. Community surveillance and response to maternal and child deaths in low- and middle-income countries: A scoping review. PLoS One. 2021;16(3):e0248143.

11. Rahman AE, Perkins J, Islam S, Siddique AB, Moinuddin M, Anwar MR, et al. Knowledge and involvement of husbands in maternal and newborn health in rural Bangladesh. BMC Pregnancy and Childbirth. 2018 Jun 18;18(1):247.

12. Kebede AA, Gessesse DN, Aklil MB, Temesgan WZ, Abegaz MY, Anteneh TA, et al. Low husband involvement in maternal and child health services and intimate partner violence increases the odds of postpartum depression in northwest Ethiopia: A community-based study. PLoS One. 2022;17(10):e0276809.

13. Sharma N, Shastri S, Shastri S. Does urbanization level and types of urban settlements matter for child stunting prevalence in India? Empirical evidence based on nighttime lights data. Cities. 2023 Sep 1;140:104388.

14. Khan MdN, Kumar P, Rahman MdM, Islam Mondal MdN, Islam MM. Inequalities in Utilization of Maternal Reproductive Health Care Services in Urban Bangladesh: A Population-Based Study. SAGE Open. 2020 Jan 1;10(1):2158244020914394.

15. Greene S, Vaccaro M, Bernier A, Griffith G, Ion A, Maurice R, et al. “Digging in”: stigma and surveillance in the lives of pregnant and breastfeeding mothers who consume cannabis. Critical Public Health. :1–12.

16. Mulikaburt T, Apidechkul T, Wongnuch P, Kitchanapaibul S, Udplong A, Srichan P, et al. Components of stigma and its impact on maternal and child health service and outcomes: perspective of Akha hill tribe women in Thailand. BMC Health Services Research. 2022 Oct 19;22(1):1263.

17. Nichols TR, Welborn A, Gringle MR, Lee A. Social Stigma and Perinatal Substance Use Services: Recognizing the Power of the Good Mother Ideal. Contemporary Drug Problems. 2021 Mar 1;48(1):19–37.

18. Roberts BW, Yao J, Trzeciak CJ, Bezich LS, Mazzarelli A, Trzeciak S. Income Disparities and Nonresponse Bias in Surveys of Patient Experience. J Gen Intern Med. 2020 Jul;35(7):2217–8.

19. Jang M, Vorderstrasse A. Socioeconomic Status and Racial or Ethnic Differences in Participation: Web-Based Survey. JMIR Res Protoc. 2019 Apr 10;8(4):e11865.

20. Van Loon AJM, Tijhuis M, Picavet HSJ, Surtees PG, Ormel J. Survey non-response in the Netherlands: effects on prevalence estimates and associations. Ann Epidemiol. 2003 Feb;13(2):105–10.

21. Diese M, Kalonji A, Izale B, Villeneuve S, Kintaudi NM, Clarysse G, et al. Community-based maternal, newborn, and child health surveillance: perceptions and attitudes of local stakeholders towards using mobile phone by village health volunteers in the Kenge Health Zone, Democratic Republic of Congo. BMC Public Health. 2018 Mar 5;18(1):316.

22. Verity C, Nicoll A. Consent, confidentiality, and the threat to public health surveillance. BMJ. 2002 May 18;324(7347):1210–3.

23. Kaweenuttayanon N, Pattanarattanamolee R, Sorncha N, Nakahara S. Community surveillance of COVID-19 by village health volunteers, Thailand. Bull World Health Organ. 2021 May 1;99(5):393–7.

24. Nyagah LM, Bangura S, Omar OA, Karanja M, Mirza MA, Shajib H, et al. The importance of community health workers as frontline responders during the COVID-19 pandemic, Somalia, 2020-2021. Front Public Health. 2023;11:1215620.

25. Owusu L, Tuwor RD, Ackam N, Loglo A, Agbavor B, Agbanyo A, et al. Role and capacity needs of community based surveillance volunteers in the integrated management of skin neglected tropical diseases (skin NTDs): a qualitative study from central Ghana. BMC Public Health. 2023 Jun 6;23(1):1086.

26. Nontapet O, Maneerattanasak S, Jaroenpool J, Phumee A, Krachai W, Napet P, et al. Understanding dengue solution and larval indices surveillance system among village health volunteers in high- and low-risk dengue villages in southern Thailand. One Health. 2022 Dec;15:100440.

27. Sunguya BF, Mlunde LB, Ayer R, Jimba M. Towards eliminating malaria in high endemic countries: the roles of community health workers and related cadres and their challenges in integrated community case management for malaria: a systematic review. Malar J. 2017 Jan 3;16(1):10.

28. Dil Y, Strachan D, Cairncross S, Korkor AS, Hill Z. Motivations and challenges of community-based surveillance volunteers in the northern region of Ghana. J Community Health. 2012 Dec;37(6):1192–8.

29. Sakeah E, Aborigo RA, Debpuur C, Nonterah EA, Oduro AR, Awoonor-Williams JK. Assessing selection procedures and roles of Community Health Volunteers and Community Health Management Committees in Ghana's Community-based Health Planning and Services program. PLoS One. 2021;16(5):e0249332.

30. Bezbaruah S, Wallace P, Zakoji M, Padmini Perera WL, Kato M. Roles of community health workers in advancing health security and resilient health systems: emerging lessons from the COVID-19 response in the South-East Asia Region. WHO South-East Asia Journal of Public Health [Internet]. 2021;10(Suppl 1). Available from: https://journals.lww.com/wsep/fulltext/2021/10001/roles_of_community_health_workers_in_advancing.9.aspx

## Table 1. Descriptive Characteristics of Study Participants [Quantitative Survey; n=601; 50% Men]

| Characteristics | | Men | | Women | | Total | |
|---|---|---|---|---|---|---|---|
| | | n | % | n | % | n | % |
| Relationship with Head of Household | | | | | | | |
| | Head of household | 247 | 81,9 | 15 | 4,9 | 261 | 43,5 |
| | Spouse of the head of household | 2 | 0,6 | 262 | 87,5 | 264 | 43,9 |
| | Son/Daughter | 36 | 12,1 | 12 | 3,9 | 48 | 8 |
| | Other (Parent/Brother/Sister/In-Law, etc) | 16 | 5,4 | 11 | 3,7 | 28 | 4,6 |
| Marital Status | | | | | | | |
| | Single/Unmarried | 36 | 12 | 13 | 4,3 | 49 | 8,2 |
| | Married | 257 | 85,2 | 270 | 90,2 | 527 | 87,7 |
| | Divorced/Separated | 8 | 2,8 | 17 | 5,5 | 25 | 4,1 |
| Education | | | | | | | |
| | <6 years of education | 1 | 0,2 | 5 | 1,8 | 6 | 1 |
| | 6-12 years of primary education | 40 | 13,4 | 57 | 19,2 | 97 | 16,3 |
| | 12 years of education (high school graduate) | 162 | 53,6 | 139 | 46,3 | 300 | 49,9 |
| | College/University | 99 | 32,8 | 99 | 32,8 | 197 | 32,8 |
| Occupation | | | | | | | |
| | Housewife/Stay-at-home mother | 0 | 0 | 201 | 67 | 201 | 33,4 |
| | Civil servant/government employee | 19 | 6,2 | 7 | 2,4 | 26 | 4,2 |
| | Private employee | 66 | 22 | 17 | 5,7 | 83 | 13,9 |
| | Farmer/Fisher | 1 | 0,4 | 0 | 0 | 1 | 0,2 |
| | Entrepreneur/Merchant | 54 | 17,9 | 32 | 10,6 | 86 | 14,3 |
| | Self-employed | 42 | 14,2 | 6 | 1,8 | 48 | 8 |
| | Student | 14 | 4,5 | 8 | 2,8 | 22 | 3,6 |
| | Unemployed | 27 | 8,9 | 4 | 1,4 | 31 | 5,2 |
| | Retired | 41 | 13,7 | 8 | 2,8 | 50 | 8,2 |
| | Other | 37 | 12,2 | 17 | 5,5 | 53 | 9 |
| Smoking Status | | | | | | | |
| | Never smoke | 118 | 39,3 | 297 | 99 | 415 | 69,1 |
| | Smoking within this month | 147 | 48,7 | 2 | 0,8 | 149 | 24,8 |
| | Smoking within this year | 4 | 1,4 | 0 | 0 | 4 | 0,7 |
| | Smoking last year or earlier | 32 | 10,6 | 1 | 0,2 | 32 | 5,4 |

**Table 2. Quantitative Analysis: Respondent's Knowledge of Maternal and Child Health**

| | Men (n=301) | | Women (n=300) | | Total | |
|---|---|---|---|---|---|---|
| | n | % | n | % | n | % |
| In your opinion, what are the warning signs of pregnancy? | | | | | | |
| Prolonged contraction | 29 | 9,6 | 26 | 8,6 | 55 | 9,1 |
| Bleeding | 106 | 35 | 138 | 46,1 | 244 | 40,5 |
| High fever | 26 | 8,7 | 24 | 7,9 | 50 | 8,3 |
| Convulsions | 3 | 1 | 9 | 2,9 | 12 | 1,9 |
| Fetal malpresentation/malposition | 10 | 3,2 | 5 | 1,5 | 14 | 2,4 |
| Leg edema | 13 | 4,3 | 33 | 11,1 | 46 | 7,7 |
| Loss of consciousness/fainting | 7 | 2,3 | 6 | 2,1 | 13 | 2,2 |
| Difficulty in breathing | 8 | 2,7 | 25 | 8,4 | 33 | 5,6 |
| Fatigue | 16 | 5,2 | 30 | 10,1 | 46 | 7,6 |
| Hypertension | 32 | 10,5 | 80 | 26,7 | 112 | 18,6 |
| Early loss of amniotic fluid | 18 | 5,9 | 30 | 10,1 | 48 | 8 |
| Decreased fetal movements | 17 | 5,6 | 22 | 7,2 | 38 | 6,4 |
| Persistent vomiting and loss of appetite | 15 | 4,9 | 35 | 11,5 | 49 | 8,2 |
| Other | 66 | 21,8 | 104 | 34,6 | 169 | 28,2 |
| I don’t know | 103 | 34,2 | 32 | 10,7 | 135 | 22,5 |
| In your opinion, what are the warning signs during childbirth? | | | | | | |
| Early rupture of the amniotic sac | 58 | 19,1 | 93 | 31,2 | 151 | 25,1 |
| Massive bleeding during and after delivery | 101 | 33,7 | 155 | 51,8 | 257 | 42,7 |
| High fever | 1 | 0,4 | 6 | 2,2 | 8 | 1,3 |
| Prolonged contraction/labor | 29 | 9,7 | 36 | 12,1 | 66 | 10,9 |
| Loss of consciousness/fainting | 7 | 2,4 | 10 | 3,3 | 17 | 2,9 |
| Convulsions | 6 | 2 | 7 | 2,4 | 13 | 2,2 |
| Retention of placenta | 0 | 0 | 0 | 0 | 0 | 0 |
| Intrauterine fetal death | 6 | 2,1 | 7 | 2,2 | 13 | 2,1 |
| Hypertension | 37 | 12,4 | 94 | 31,4 | 131 | 21,9 |
| Greenish and foul-smelled amniotic fluid | 5 | 1,5 | 8 | 2,8 | 13 | 2,2 |
| Mother’s anxiety or intense pain | 9 | 3,2 | 6 | 2 | 15 | 2,6 |
| Mother’s inability to push during labor | 13 | 4,2 | 12 | 3,9 | 24 | 4 |
| Fetal entanglement in the umbilical cord | 19 | 6,3 | 12 | 3,9 | 31 | 5,1 |
| Other | 27 | 9 | 58 | 19,5 | 85 | 14,2 |
| I don’t know | 106 | 35,2 | 39 | 12,9 | 145 | 24,1 |
| In your opinion, what are the warning signs in postpartum mothers? | | | | | | |
| Massive bleeding | 84 | 27,7 | 155 | 51,7 | 238 | 39,7 |
| Loss of consciousness/fainting | 2 | 0,8 | 9 | 3,1 | 12 | 1,9 |
| Convulsions | 2 | 0,8 | 4 | 1,4 | 6 | 1,1 |
| High Fever | 15 | 5,1 | 18 | 6,2 | 34 | 5,6 |
| Foul-smelled vaginal mucus | 4 | 1,2 | 6 | 1,9 | 9 | 1,6 |
| Breast pain | 1 | 0,4 | 13 | 4,5 | 15 | 2,4 |
| Sadness or depressed feeling | 9 | 2,9 | 25 | 8,3 | 34 | 5,6 |
| Other | 38 | 12,7 | 73 | 24,5 | 112 | 18,6 |
| I don’t know | 170 | 56,6 | 75 | 25 | 245 | 40,8 |
| In your opinion, what are the warning signs in newborn infants? | | | | | | |
| Pale-colored feces | 1 | 0,4 | 1 | 0,4 | 2 | 0,4 |
| High fever | 38 | 12,5 | 63 | 21 | 101 | 16,7 |
| Diarrhea | 4 | 1,4 | 16 | 5,2 | 20 | 3,3 |
| Vomiting | 2 | 0,6 | 5 | 1,6 | 6 | 1,1 |
| Jaundiced eyes and skin | 125 | 41,5 | 200 | 66,6 | 325 | 54 |
| Low body temperature/hypothermia | 4 | 1,4 | 1 | 0,2 | 5 | 0,8 |
| Skin appeared blue | 20 | 6,6 | 36 | 11,9 | 56 | 9,3 |
| Small-born/low birth weight | 47 | 15,7 | 58 | 19,5 | 106 | 17,6 |
| Difficulty breathing | 45 | 15 | 50 | 16,8 | 96 | 15,9 |
| Not crying | 55 | 18,2 | 61 | 20,5 | 116 | 19,3 |
| Grunting | 4 | 1,2 | 1 | 0,4 | 5 | 0,8 |
| Convulsions | 15 | 5,1 | 31 | 10,3 | 46 | 7,7 |
| Unwilling to be fed | 30 | 9,8 | 35 | 11,6 | 64 | 10,7 |
| Redness, foul odor, or pus in the umbilical cord | 4 | 1,2 | 12 | 4,1 | 16 | 2,7 |
| Other | 43 | 14,4 | 49 | 16,4 | 93 | 15,4 |
| I don’t know | 70 | 23,2 | 21 | 7 | 91 | 15,1 |

**Table 3. Barriers and Opportunities in Establishing Community-Based Surveillance on Maternal and Child Health [Qualitative Analysis]**

| | | Respondents | Statements from Interviews/Focus Group Discussions |
|---|---|---|---|
| Barriers | High mobility of incoming and outgoing migrant workers with their children | Subdistrict Official | "Many incoming migrants, mostly those who rent houses temporarily, are sometimes unnoticed and unreported to the head of the neighbourhood. Delayed care for migrant pregnant women and children sometimes happens because of this, as they do not report themselves to the local officials, community leaders, or social health workers. Thus, they become unregistered at the Integrated Health Service Post. I think the head of the villages should go door-to-door to the rented houses to monitor." |
| | Lack of capacity of the health volunteers | Medical Staff from the Community Health Center | "Not all volunteers are digitally adept, particularly the older ones. That is our difficulty when introducing the online surveillance system with Google Forms, as only the young volunteers can do the report via Google Forms."<br>"Another challenge that we met is human error in stunting measurement. We sometimes observed that the health volunteers did not weigh the children in a correct manner, despite training had been provided to them so that they could measure the weight and height of the children correctly and accurately. " |
| | Stigma related to sensitive maternal and child health issues | Health Volunteer | "What we often find is that the parents are reluctant if health issues of their children are known by other people, especially those issues that the public sees as a shame, for example, if their children are malnourished or stunting." |
| | Late response from healthcare providers | Head of Village/Neighbourhood | "If community-based surveillance is about to be implemented, strengthening intersectoral coordination is extremely needed, starting from the village level until the subdistrict, district, and Community Health Center, to ensure follow-up from community reporting is handled promptly. Nowadays, the coordination is still lacking, and the delayed response from the relevant agencies leads to the community becoming reluctant to be involved in the subsequent government programs." |
| | Unwillingness to participate in families with middle-high socioeconomic status | Health Volunteers | "There is a challenge in reaching the residents who live in exclusive neighbourhoods, as they are generally very difficult to be visited. We often got rejected when we house visited because they said that they were very busy and always working. They are also, in general, more reserved or less sociable. "<br>"Residents with middle-high income socioeconomic status who live in exclusive neighbourhoods are generally less involved in community activities. Economically, they have the means to access private healthcare services, and every time health volunteers or staff from the Community Health Center come to their house, they are always difficult to be met. We have to repeat the visit multiple times." |

| | | | |
|---|---|---|---|
| **Opportunities** | Community's positive perceptions of surveillance | Head of Village/Neighbourhood | "In general, the community is open to community-based surveillance. In many instances, community members even have a high awareness of self-reporting their condition, as they know it is needed to make regulations related to healthcare services." |
| | Existing community empowerment activities | District Official, Health Volunteers | "Currently, the 'Blessed Friday' program is regularly conducted in all villages. There, food products such as vegetables, meat, rice, and other things are given to those in need for free."<br>"In the Qurán recitation routine events, the religious leaders and health volunteers always remind the pregnant women and small children to visit POSYANDU and the elderly to go to the POSBINDU."*<br>"If there is a person who is ill, others chip in to help, to visit the ill person, and jointly collect money to give a little financial help to the ill if they are hospitalized. This activity happens often and easily because we often meet each other during the weekly Qurán recitations." |
| | Active role of health volunteers | Subdistrict Official, Community Member | "The health volunteers are completely social-based, with no salary at all, nothing. The city's health office often tells us they have budget limitations to conduct community activities, but the volunteers are OK with that. I greatly respect the volunteers as they are truly social and never ask about transportation costs when doing their activities."<br>"Despite all limitations, the health volunteers always actively do their activities every Friday. They conduct house visits, or what we call "sweeping", in their neighbourhoods to identify if there are new pregnant women in the community. They also always diligently remind pregnant women to check their condition at the Community Health Center, or mothers with small children to visit the POSYANDU." |
| | Potential collaboration between stakeholders and community organisations | Medical Staff from the Community Health Center, Health Volunteers | "The 'Blessed Friday' program may also invite charity organizations or private industries via their CSR to participate as donors in providing free foods for those in need in every village. The health office or the city's mayor can help coordinate to involve these donors".<br>"To be able to implement a digital-based surveillance system in the community widely, we will need the involvement from the youth organization as they have bright ideas and are smart. However, many of them have little time as most are still in school or have a day job."<br>"Another institution that is very important to be involved is the academics from universities. For example, the residents are very excited and enthusiastic every time students from Universitas Indonesia come to provide public health education to the community." |
| | Digital communication channels | District Official, Head of Village/Neighbourhood | "The communication between health volunteers and the Community Health Center is going well. To communicate with each other, there is a WhatsApp group between all volunteers in the district and subdistricts with the medical staff at the Community Health Center. If there is an emergency, the volunteers can contact the Community Health Center immediately to obtain help."<br>"We have a WhatsApp group with all heads of households in the village for means of coordination. For example, during COVID, if there is news of a community member testing positive, the news is shared so everybody directly knows." |

*Abbreviation: POSYANDU (*Pos Pelayanan Terpadu*; The Integrated Health Service Post). POSBINDU (*Pos Pembinaan Terpadu; The Integrated Guidance Post for Noncommunicable Diseases).*

**Supplemental Table 1. Quantitative Analysis: Respondent's Attitudes on Maternal and Child Health**

| | Men (n=301) | | Women (n=300) | | Total | |
|---|---|---|---|---|---|---|
| | **n** | **%** | **n** | **n** | **%** | **n** |
| Health monitoring for mothers and children is not obligatory to be implemented. | | | | | | |
| Very Agree | 2 | 0,6 | 3 | 1 | 5 | 0,8 |
| Agree | 14 | 4,8 | 21 | 7 | 35 | 5,9 |
| Disagree | 261 | 86,6 | 263 | 87,7 | 524 | 87,1 |
| Very Disagree | 24 | 8,1 | 13 | 4,3 | 37 | 6,2 |
| Maternal blood tests will help check the health condition of pregnant mothers. | | | | | | |
| Very Agree | 19 | 6,2 | 17 | 5,7 | 36 | 6 |
| Agree | 276 | 91,5 | 282 | 94,1 | 557 | 92,8 |
| Disagree | 7 | 2,4 | 1 | 0,2 | 8 | 1,3 |
| Very Disagree | 0 | 0 | 0 | 0 | 0 | 0 |
| Maternal blood tests will help in preparing blood donors if complications occur. | | | | | | |
| Very Agree | 23 | 7,5 | 19 | 6,2 | 41 | 6,9 |
| Agree | 275 | 91,3 | 280 | 93,6 | 556 | 92,4 |
| Disagree | 4 | 1,2 | 1 | 0,2 | 4 | 0,7 |
| Very Disagree | 0 | 0 | 0 | 0 | 0 | 0 |
| Public health education related to maternal and child health is the obligation of health volunteers. | | | | | | |
| Very Agree | 15 | 5,1 | 8 | 2,8 | 24 | 4 |
| Agree | 215 | 71,5 | 222 | 74,3 | 438 | 72,9 |
| Disagree | 70 | 23,4 | 69 | 23 | 139 | 23,2 |
| Very Disagree | 0 | 0 | 0 | 0 | 0 | 0 |
| Childbirth should be assisted by trained medical professionals such as midwives and doctors | | | | | | |
| Very Agree | 45 | 14,8 | 33 | 11,2 | 78 | 13 |
| Agree | 251 | 83,4 | 266 | 88,8 | 518 | 86,1 |
| Disagree | 5 | 1,7 | 0 | 0 | 5 | 0,9 |
| Very Disagree | 0 | 0 | 0 | 0 | 0 | 0 |
| Pregnancy, childbirth, postpartum, and children's growth will go smoothly if local myths are followed. | | | | | | |
| Very Agree | 1 | 0,2 | 1 | 0,2 | 1 | 0,2 |
| Agree | 42 | 13,9 | 38 | 12,8 | 80 | 13,4 |
| Disagree | 244 | 81 | 251 | 83,8 | 495 | 82,4 |
| Very Disagree | 15 | 4,9 | 9 | 3,2 | 24 | 4 |
| It is no problem if childbirth is assisted by a shaman/traditional healer. | | | | | | |
| Very Agree | 0 | 0 | 0 | 0 | 0 | 0 |
| Agree | 126 | 41,7 | 93 | 31 | 218 | 36,3 |
| Disagree | 164 | 54,4 | 196 | 65,5 | 360 | 59,9 |
| Very Disagree | 12 | 3,9 | 11 | 3,6 | 23 | 3,8 |

**Supplemental Table 2. Quantitative Analysis: Where to Report When Health Issues are Detected in Mothers and Children (n=601; 50% men)**

| | Pregnant Mothers | | | Delivering Mothers | | | Postpartum Mothers | | | Newborn Infants | | |
|---|---|---|---|---|---|---|---|---|---|---|---|---|
| | Men | Women | Total | Men | Women | Total | Men | Women | Total | Men | Women | Total |
| | % | % | % | % | % | % | % | % | % | % | % | % |
| When there is an alarming health issue in pregnant mothers/delivering mothers/postpartum mothers/newborn infants, where should we report it? | | | | | | | | | | | | |
| City’s Health Office | 0,6 | 0,2 | 0,4 | 0 | 0 | 0 | 0 | 0 | 0 | 0 | 0 | 0 |
| Hospital | 48 | 38,9 | 43,5 | 53,2 | 42,7 | 48 | 51,2 | 39,8 | 45,5 | 52,5 | 42,5 | 47,5 |
| Community Health Center | 47,8 | 42,5 | 45,2 | 45,4 | 40,8 | 43,1 | 46,1 | 39,9 | 43 | 48,5 | 41,4 | 45 |
| Clinic | 23,2 | 18,6 | 20,9 | 24,7 | 17 | 20,9 | 24,7 | 18,3 | 21,5 | 24,3 | 20,5 | 22,4 |
| Head of Village | 13,5 | 14,8 | 14,1 | 11,5 | 13,8 | 12,6 | 11,8 | 11,9 | 11,8 | 10,7 | 11,4 | 11 |
| Medical Professional | 9,7 | 16,1 | 12,9 | 10,4 | 13,5 | 12 | 9 | 15,6 | 12,3 | 9,3 | 13,9 | 11,6 |
| Public Official (District/Subdistrict) | 0 | 0 | 0 | 0 | 0 | 0 | 0 | 0 | 0 | 0 | 0,2 | 0,1 |
| Community Leader/Religious Leader | 0,2 | 0 | 0,1 | 0 | 0 | 0 | 0 | 0 | 0 | 0 | 0 | 0 |
| Health Volunteer | 13,3 | 12,7 | 13 | 11,9 | 11,3 | 11,6 | 11,3 | 9,3 | 10,3 | 11,1 | 9,3 | 10,2 |
| Family | 1,4 | 1,2 | 1,3 | 1,5 | 1,6 | 1,5 | 2,1 | 1,2 | 1,6 | 1,5 | 1 | 1,3 |
| Other | 2 | 3,5 | 2,7 | 1 | 2,6 | 1,8 | 0,8 | 3 | 1,9 | 0,8 | 2,8 | 1,8 |
| I do not report | 2,2 | 3,3 | 2,7 | 1,8 | 2,8 | 2,3 | 1,8 | 2,8 | 2,3 | 1,8 | 2,8 | 2,3 |
| I observe no cases | 4,4 | 3,3 | 3,9 | 4,6 | 3,7 | 4,2 | 5,2 | 5,6 | 5,4 | 5,2 | 5 | 5,1 |
| I don’t know if there are cases | 0,8 | 1,4 | 1,1 | 1,2 | 1,5 | 1,4 | 0,8 | 1,4 | 1,1 | 0,6 | 0,8 | 0,7 |
| I don’t know where to report | 2,3 | 1,6 | 1,9 | 2,3 | 0,4 | 1,4 | 2,5 | 0,6 | 1,6 | 2,5 | 0,4 | 1,5 |
| To your knowledge, are there feedback/responses from any relevant agencies regarding the report of health issues? | | | | | | | | | | | | |
| Yes | 87,4 | 92,6 | 90 | 86,6 | 92,2 | 89,4 | 87 | 89,9 | 88,5 | 87,6 | 92 | 89,8 |
| No | 0 | 0 | 0 | 1,2 | 1 | 1,1 | 0,6 | 1 | 0,8 | 0 | 0,6 | 0,3 |
| I don’t know | 12,6 | 7,4 | 10 | 12,2 | 6,8 | 9,5 | 12,4 | 9,1 | 10,8 | 12,4 | 7,4 | 9,9 |

**Supplemental Table 3. Quantitative Analysis: Perceptions Towards Healthcare Facilities (n=601; 50% men)**

| | Pregnant Mothers | | | Postpartum Mothers | | | Newborn Infants | | |
|---|---|---|---|---|---|---|---|---|---|
| | Men | Women | Total | Men | Women | Total | Men | Women | Total |
| | % | % | % | % | % | % | % | % | % |
| Do you find difficulties in reporting health cases related to pregnant mothers/postpartum mothers/newborn infants? | | | | | | | | | |
| Yes | 0,8 | 1,2 | 1 | 1,6 | 1,2 | 1,4 | 1,4 | 1,4 | 1,4 |
| No | 79,7 | 86 | 82,8 | 78,3 | 86,2 | 82,2 | 78,1 | 85,4 | 81,7 |
| Not Sure | 19,6 | 12,8 | 16,2 | 20,1 | 12,6 | 16,4 | 20,6 | 13,2 | 16,9 |
| Do you find difficulties in obtaining healthcare services for pregnant mothers/postpartum mothers/newborn infants? | | | | | | | | | |
| Yes | 1,5 | 1,6 | 1,6 | 1,5 | 1 | 1,3 | 1,4 | 1,4 | 1,4 |
| No | 79,5 | 86,4 | 82,9 | 79,1 | 87 | 83 | 78,8 | 86 | 82,4 |
| Not Sure | 19 | 12 | 15,5 | 19,4 | 12 | 15,7 | 19,8 | 12,6 | 16,2 |

**Supplemental Table 4. Quantitative Analysis: Monitoring of Mothers' and Children's Health (n=601; 50% men)**

| | Pregnant Mothers | | | Delivering/Postpartum Mothers | | | Newborn Infants | | |
|---|---|---|---|---|---|---|---|---|---|
| | Men | Women | Total | Men | Women | Total | Men | Women | Total |
| | % | % | % | % | % | % | % | % | % |
| Would you allow the community to monitor the health of pregnant/delivering/postpartum mothers and newborn infants in your area? | | | | | | | | | |
| Yes | 96,9 | 99 | 98 | 96,3 | 99,6 | 98 | 97,1 | 99,6 | 98,3 |
| No | 3,1 | 1 | 2 | 3,7 | 0,4 | 2 | 2,9 | 0,4 | 1,7 |
