# Supplementary material for: Barriers and opportunities in developing community-based maternal and child health surveillance: A mixed methods study in Depok, Indonesia
Source: PLoS One. 2025 Nov 17;20(11):e0332469. doi: 10.1371/journal.pone.0332469 (PMC12622817; doi:10.1371/journal.pone.0332469)
Supplement: Attachment — Submitted filename: Review and Comments_RA.docx [file pone.0332469.s006.docx]

Study Title: Barriers and opportunities in developing community based maternal and child health surveillance: a mixed methods study in Depok, Indonesia

**Summary of the research:** This research study titled “Barriers and opportunities in developing community based maternal and child health surveillance: a mixed methods study in Depok, Indonesia,” explores the factors influencing the effectiveness of a community-based surveillance system for maternal and child health in Depok, Indonesia. The study employs a mixed method approach combining quantitative data from 300 households and qualitative insights from interviews and focus group discussions with various stakeholders. The findings are beneficial for the improvement of the already established community health services in Indonesia. In this regard the key findings include:

1. A significant proportion of the population lacks knowledge of warnings signs during pregnancy, childbirth and newborn care, with men showing higher ignorance rates than women.
2. Most respondents support community health monitoring
3. Barriers identified include high mobility of migrant families, inadequate capacity of health volunteers, stigma, delayed healthcare responses.
4. Opportunities include positive community perception of surveillance, ongoing community empowerment activities, active roles of health volunteers, potential stakeholder collaboration, and the use of digital communication channels.

The study concludes that public health education targeting men is crucial and addressing the identified barriers could help establish sustainable community-based surveillance system for maternal and child health.

**Examples and evidence**

**Major issues**: I did not find any major issues on the study method and data analysis.

The methods used in the study appear to be appropriate and well suited for the research objectives. The mixed-method approach allows for the collection of broad statistical data and in-depth insights from stakeholders.

*Quantitative data collection:* The use of an interviewer assisted questionnaire in 300 households across three subdistricts ensures a substantial sample size for statistical analysis. The selection of random sampling method enhances the representativeness of the sample.

*Qualitative data collection:* In-depth interviews and focus group discussions with a diverse range of stakeholders enriched the qualitative data collection. It helps in understanding the nuanced barriers and opportunities from multiple perspectives.

*Data analysis:* the use of weighted analysis for quantitative data and thematic analysis for qualitative data ensures that the findings are robust and well analyzed.

**Minor issues:** I suggest that the authors provide in a table format or otherwise some characteristics of the various respondents chosen for the in-depth interviews such as their age group, years of service in their respective positions. For example, break down the years of service to less than 3 years, between 3-5 years, 5-10 years, and greater than 10 years. This information would add value to the information provided.

In addition, a description of the composition of the participants in the FGDs and their age range, how many men and women participated in the FGDs. It would enable the readers to get an understanding of the value of the data collected.

**Discussion Section:**

**Major issues**: the authors cover a lot of valuable insight and information in this study. I feel the discussion can be improved by restructuring it. Here are some suggestions to improve it.

*Elaborate on the key findings*. Highlight the most important findings prominently. For instance, the disparity in knowledge between men and women and high approval rate of monitoring could be emphasized.

*Address barriers and opportunities:* discuss each barrier and opportunity in a bit more derail. Use quotes from in-depth interviews and FGDs to illustrate the point.

*Comparison with previous studies*: When comparing your findings with previous studies, provide specific references and context and explain why the findings in this study align or differ from previous studies and what this means for your study.

*Recommendations:* Offer clear and actionable recommendations based on your findings. For example, the authors could suggest targeted public health education programs for men.

*Conclusion:* summarize the key points and emphasize the importance of this study.

Other points:

1. I found that the authors have not described any limitations they had in planning and doing the study. I suggest providing a paragraph on what the authors feel might be the limitations of this study would strengthen the manuscript.
2. In supplement table-1, I suggest instead of the options “very agree” and “very disagree”, these be stated as “*fully agree*” and “*fully disagree*”
3. An overall editing of the English is recommended.
