# Supplementary material for: Barriers and opportunities in developing community-based maternal and child health surveillance: A mixed methods study in Depok, Indonesia
Source: PLoS One. 2025 Nov 17;20(11):e0332469. doi: 10.1371/journal.pone.0332469 (PMC12622817; doi:10.1371/journal.pone.0332469)
Supplement: Attachment — Submitted filename: renamed_09292.docx [file pone.0332469.s008.docx]

Comments – PONE-D-24-59311-R1

**Title** - Barriers and Opportunities in Developing Community-Based Maternal and Child Health

Surveillance: A Mixed Methods Study in Depok, Indonesia

The title is appropriate

**Abstract** – Abstract is well structured but the section sub-titled as “Discussion” should be changed to “Conclusion”. Key words provided

**Introduction** – Statement of problem is well written. The magnitude of the problem, rationale for the study and study objectives are well presented.

**Methods** – The specific name of the study design is not mentioned. What is mentioned is the approach. This appears to be a descriptive cross-sectional study with a mixed methods approach.

- Study setting is well described.
- The minimum sample size estimation for the quantitative part of the study not calculated. appropriately written.
- What type of random selection was used to select the households?
- Data collection and analysis are well written
- Provide information on the number of interviews and FGDs conducted.

**Result** – the findings are fairly well presented.

- *…….. disproportionately higher in men (34.2%) than women (10.7%).* - Is this difference found to be statistically significant? This should be analysed
- *……… disproportionate proportion between men (35.2%) and women (12.9%).* - Is this difference found to be statistically significant? This should be analysed
- The qualitative findings should be improved upon by inserting some appropriate quotes in the various sub-themes even though this is on table 3.

**Discussion –** Findings fairly well discussed.

There is no conclusion section after the discussion. The study limitations were not mentioned.

**References** - adequate
