# Supplementary material for: Barriers and opportunities in developing community-based maternal and child health surveillance: A mixed methods study in Depok, Indonesia
Source: PLoS One. 2025 Nov 17;20(11):e0332469. doi: 10.1371/journal.pone.0332469 (PMC12622817; doi:10.1371/journal.pone.0332469)
Supplement: Attachment — Submitted filename: Comments to the Reviewers.docx [file pone.0332469.s009.docx]

*We thank the three reviewers for the overall positive feedback and support of our manuscript, and the editors for the opportunity to submit an improved manuscript. We are grateful for the insightful comments and constructive suggestions, which have helped us improve the quality of our work. Please find below our point-by-point response addressing the feedback from the reviewers.*

**Reviewer's Responses to Questions**

1. Is the manuscript technically sound, and do the data support the conclusions? The manuscript must describe a technically sound piece of scientific research with data that supports the conclusions. Experiments must have been conducted rigorously, with appropriate controls, replication, and sample sizes. The conclusions must be drawn appropriately based on the data presented.

Reviewer #1: Yes

Reviewer #2: Yes

Reviewer #3: Yes

2. Has the statistical analysis been performed appropriately and rigorously?

Reviewer #1: Yes

Reviewer #2: Yes

Reviewer #3: Yes

3. Have the authors made all data underlying the findings in their manuscript fully available? The PLOS Data policy requires authors to make all data underlying the findings described in their manuscript fully available without restriction, with rare exception (please refer to the Data Availability Statement in the manuscript PDF file). The data should be provided as part of the manuscript or its supporting information, or deposited to a public repository. For example, in addition to summary statistics, the data points behind means, medians and variance measures should be available. If there are restrictions on publicly sharing data—e.g., participant privacy or use of data from a third party—those must be specified.

Reviewer #1: Yes

Reviewer #2: Yes

Reviewer #3: Yes

4. Is the manuscript presented in an intelligible fashion and written in standard English? PLOS ONE does not copyedit accepted manuscripts, so the language in submitted articles must be clear, correct, and unambiguous. Any typographical or grammatical errors should be corrected at revision, so please note any specific errors here.

Reviewer #1: Yes

Reviewer #2: Yes

Reviewer #3: Yes

5. Review Comments to the Author

**Reviewer #1**: The manuscript is neat and well prepared, I would have only suggested that the discussion may need to be summarized little bit as there are many details from the results are repeated here, it may be better to point out the important findings and directly connect that with other studies. Some sentences like this "We observed that around 15.1%-24.1% of the general public appeared to have insufficient knowledge" need to be corrected, instead of "general public" it is suitable to say "study population". At the abstract you mentioned "Men exhibited higher ignorance rates (34.2%, 35.2%, 23.2%) than women (10.7%,12.9%, 7.0%).", I would suggest that the you change the word "ignorance" as this definition is broad and cannot be determined subject wise. Maybe you could use "less knowledgeable to the subject matter..."

*We thank Reviewer #1 for their constructive feedback on our manuscript. In response to the suggestion, we have summarized the Discussion while retaining its most important information. Additionally, we created a Conclusion section at the end of the Discussion to highlight the main findings and propose our recommendation to the field.* ***Line 429 in the revised manuscript, highlighted in yellow***

***Line 347 in the revised manuscript, highlighted in yellow***

*For the sentence "We observed that around 15.1%-24.1% of the general public appeared to have insufficient knowledge of …", we have now amended the sentence into "We observed that around 15.1%-24.1% of the* ***study population*** *appeared to have insufficient knowledge of …"*

***Line 41 in the revised manuscript, highlighted in yellow***

*For the sentence “Men exhibited higher ignorance rates (34.2%, 35.2%, 23.2%) than women (10.7%,12.9%, 7.0%)”, we have also revised the sentence into “Men appeared to be* ***less knowledgeable on these matters*** *(34.2%, 35.2%, 23.2%) than women (10.7%,12.9%, 7.0%)." in accordance with the reviewer's recommendation.*

**Reviewer #2**: The study explores the effectiveness of community-based surveillance system for maternal and child health in Depok, Indonesia. The methods used in the study appear to be appropriate and well suited for the research objectives. The mixed-method approach allows for the collection of broad statistical data and in-depth insights from stakeholders. The findings are beneficial for the improvement of the already established community health services in Indonesia.

**Minor issues:** I suggest that the authors provide in a table format or otherwise some characteristics of the various respondents chosen for the in-depth interviews such as their age group, years of service in their respective positions. For example, break down the years of service to less than 3 years, between 3-5 years, 5-10 years, and greater than 10 years. This information would add value to the information provided. In addition, a description of the composition of the participants in the FGDs and their age range, how many men and women participated in the FGDs. It would enable the readers to get an understanding of the value of the data collected.

*We deeply thank and appreciate Reviewer #2 for his/her overall positive response and support of our manuscript. To address the suggestion, we have now provided more details on the characteristics of the respondents or participants for the interviews and FGDs.*

***Line 165 in the revised manuscript, highlighted in yellow***

*“The characteristics of the respondents for the in-depth interviews and FGDs were described in* ***Supplemental Table 1****.”*

***(We renumbered the supplemental tables, as this table appears first in the order of the manuscript).***

***Supplemental Table 1: Characteristics of Respondents for the In-depth Interviews and Focus Group Discussions [Qualitative Analysis]***

|  |  | ***In-depth Interviews***  ***(n=23)*** | ***Focus Group Discussions***  ***(n=2)*** |
| --- | --- | --- | --- |
| *Public Officials, City Level* | *n* | *0* | *3* |
|  | *Sex (%women)* | *-* | *100* |
|  | *Job Level (%senior)* | *-* | *67* |
|  |  |  |  |
| *Public Officials, District Level* | *n* | *7^a^* | *1* |
|  | *Sex, %women* | *57* | *100* |
|  | *Job Level, %senior* | *71* | *100* |
|  |  |  |  |
| *Public Officials, Subdistrict Level* | *n* | *7^b^* | *2* |
|  | *Sex, %women* | *57* | *50* |
|  | *Job Level, %senior* | *29* | *100* |
|  |  |  |  |
| *Medical Staff* | *n* | *10^c^* | *3^e^* |
|  | *Sex, %women* | *100* | *100* |
|  | *Job Level, %senior* | *40* | *67* |
|  |  |  |  |
| *Head of Neighbourhoods* | *n* | *4^d^* | *0* |
|  | *Sex, %women* | *25* | *-* |
|  | *>10 years of leadership, %yes* | *25* | *-* |
|  |  |  |  |
| *Health Volunteers* | *n* | *3^d^* | *6^f^* |
|  | *Sex, %women* | *100* | *100* |
|  | *>10 years of service, %yes* | *67* | *67* |
|  |  |  |  |
| *Community Members* | *n* | *3^d^* | *0* |
|  | *Sex, %women* | *33* | *-* |
|  | *>10 years of residency, %yes* | *67* | *-* |

*Data are presented as percentages (%) for categorical variables. ^a^Five staff from Sukmajaya, and two staff from Cimanggis Districts; ^b^Three staff from Curug, two from Tirtajaya, and two from Mekarjaya Subdistricts; ^c^Five staff from Cimanggis, and five from Sukmajaya Community Health Centers; ^d^At least one respondent from each of Curug, Tirtajaya, and Mekarjaya Subdistricts; ^e^Two GP coordinators from Cimanggis and Sukmajaya Community Health Centers, one paediatrician from Universitas Indonesia City Referral Hospital; ^f^Two health volunteers each from Curug, Tirtajaya, and Mekarjaya Subdistricts*

**Discussion Section:**

*Elaborate on the key findings*. Highlight the most important findings prominently. For instance, the disparity in knowledge between men and women and high approval rate of monitoring could be emphasized.

*Recommendations:* Offer clear and actionable recommendations based on your findings. For example, the authors could suggest targeted public health education programs for men.

*Conclusion:* summarize the key points and emphasize the importance of this study.

*We have now incorporated these suggestions to enrich our Discussion, by creating a* ***Conclusion*** *section that highlights the* ***key findings*** *prominently and includes actionable* ***recommendations*** *for future implementation.*

***Line 429 in the revised manuscript, highlighted in yellow***

***Conclusion***

*“This study highlights the barriers and opportunities in establishing a community-based surveillance system for maternal and child health in Depok, Indonesia. We observed that while most community members support health monitoring, knowledge gaps—particularly among men—persist, and logistical challenges such as high population mobility, health volunteer capacity limitations, and social stigma hinder effective implementation. However, strong community engagement, existing empowerment programs, digital communication channels, and stakeholder collaboration offer promising opportunities. Addressing these challenges while leveraging local strengths can contribute to a sustainable, well-integrated surveillance system that improves maternal and child health outcomes. Future efforts should focus on improving public health education, particularly for men to encourage their involvement in ensuring the health of mothers and children, enhancing health volunteer training, and strengthening intersectoral coordination to ensure long-term success.”*

**Other points:**

1. I found that the authors have not described any limitations they had in planning and doing the study. I suggest providing a paragraph on what the authors feel might be the limitations of this study would strengthen the manuscript.
2. In supplement table-2, I suggest instead of the options “very agree” and “very disagree”, these be stated as “*fully agree*” and “*fully disagree*”

*We have now mentioned several* ***strengths and limitations*** *of the study, and renamed the options in Supplemental Table 2 as suggested.*

***Line 414 in the revised manuscript, highlighted in yellow***

*Strengths and Limitations of the Study*

*“This study employs a robust mixed-methods approach, integrating quantitative surveys with qualitative insights to provide a comprehensive understanding of community-based maternal and child health surveillance. The large sample size (300 households) and diverse stakeholder insights enhance representativeness and real-world* *applicability****,*** *capturing on-the-ground challenges and best practices from multiple levels of community and healthcare stakeholders, making the findings highly relevant for policy and intervention planning.”*

*“However, several limitations exist. The cross-sectional design restricts the ability to infer causal relationships between identified factors and surveillance effectiveness. Additionally, self-reported data in the household survey may be subject to recall or social desirability bias, particularly in sensitive topics such as stigma and healthcare-seeking behaviors. Moreover, while the study covers three subdistricts in Depok, its generalizability to other regions in Indonesia may be limited due to variations in health system infrastructure and socio-cultural factors. Future research should consider longitudinal studies and broader geographic coverage to strengthen applicability.”*

***Supplemental Table 2 in the revised manuscript, highlighted in yellow***

**Reviewer #3**: Title - Barriers and Opportunities in Developing Community-Based Maternal and Child Health Surveillance: A Mixed Methods Study in Depok, Indonesia. The title is appropriate.

Abstract – Abstract is well structured but the section sub-titled as “Discussion” should be changed to “Conclusion”. Key words provided.

*We thank Reviewer #3 for his/her valuable feedback,* *which have helped clarify and strengthen our manuscript. In the Abstract, we have changed the subtitle "Discussion" to "Conclusion" as recommended.* ***Line 50 in the revised manuscript, highlighted in yellow***

Introduction – Statement of problem is well written. The magnitude of the problem, rationale for the study and study objectives are well presented.

Methods – The specific name of the study design is not mentioned. What is mentioned is the approach. This appears to be a descriptive cross-sectional study with a mixed methods approach. • Study setting is well described. • The minimum sample size estimation for the quantitative part of the study not calculated. appropriately written. • What type of random selection was used to select the households? • Data collection and analysis are well written • Provide information on the number of interviews and FGDs conducted.

- *We have now stated the study design as “a descriptive cross-sectional study with a mixed methods approach” to ensure clarity**.* ***Line 124 in the revised manuscript, highlighted in yellow***
- *We have now included information on the minimum sample size estimation of the quantitative study, as suggested.*

***Line 134 in the revised manuscript, highlighted in yellow***

*“Quantitative data were collected from 300 households residing in three subdistricts in Depok (Mekarjaya, Curug, and Tirtajaya) with an interviewer-assisted questionnaire. The three subdistricts were purposively selected by the Depok City Health Office, based on their relatively higher prevalence of maternal and children health issues compared to other subdistricts. The sample size was 100 households per subdistrict, calculated based on a 95% confidence level, a 10% precision level, a 50% proportion of events, and a design effect of 1.”*

- *We have provided additional details on the sampling procedures for selecting the households.*

***Line 140 in the revised manuscript, highlighted in yellow***

*“The sampling procedure followed a two-stage cluster and random sampling method within each subdistrict to select the households. In the first stage, twenty neighbourhoods/hamlets from each of the three subdistricts were selected as the clusters using the probability proportional to size method. In the second stage, five households per hamlet were randomly selected using a computer-based random generator from the household list of each selected hamlet.”*

- *We have specified the number of interviews and FGDs conducted throughout the study, along with the respondents, stakeholders, and community representatives involved in each session (detailed in* ***Supplemental Table 1****, as in our earlier response to Reviewer #2).*

Result – the findings are fairly well presented.

• …….. disproportionately higher in men (34.2%) than women (10.7%). - Is this difference found to be statistically significant? This should be analysed.

• ……… disproportionate proportion between men (35.2%) and women (12.9%). - Is this difference found to be statistically significant? This should be analysed.

*The differences were statistically significant. We have incorporated the p-values for differences in knowledge of pregnancy, childbirth, postpartum, and newborn care between men and women in* ***Table 2.***

• The qualitative findings should be improved upon by inserting some appropriate quotes in the various sub-themes even though this is on table 3.

- *We have incorporated several direct respondent quotes from Table 3 into the Results section.*

***Line 233, 245, 252, 258, 264, 277, 288, 299, 318, 336 in the revised manuscript, highlighted in yellow***

Discussion – Findings fairly well discussed. There is no conclusion section after the discussion. The study limitations were not mentioned.

References - adequate

- *We have now outlined several limitations of our study, as well as created a Conclusion section within the Discussion (as pointed out earlier in our response to Reviewer #2)*

***Line 414 in the revised manuscript, highlighted in yellow (Study Strengths & Limitations)***

***Line 429 in the revised manuscript, highlighted in yellow (Conclusion)***
