# Supplementary material for: Barriers and opportunities in developing community-based maternal and child health surveillance: A mixed methods study in Depok, Indonesia
Source: PLoS One. 2025 Nov 17;20(11):e0332469. doi: 10.1371/journal.pone.0332469 (PMC12622817; doi:10.1371/journal.pone.0332469)
Supplement: Attachment — Submitted filename: Response to Reviewers II.docx [file pone.0332469.s010.docx]

*We thank the Academic Editor and Reviewers for their careful review and thorough feedback on our manuscript. We found the detailed comments to be constructive and have revised the manuscript accordingly. Please find below our point-by-point responses addressing each comment from the Editor and Reviewers.*

***Additional Editor Comments:***

Thank you for your resubmission of the manuscript entitled “Barriers and Opportunities in Developing Community-Based Maternal and Child Health Surveillance: A Mixed Methods Study in Depok, Indonesia.” We appreciate the thoughtful and comprehensive revisions you have made in response to reviewer feedback.

Upon further editorial review, we find your manuscript significantly improved and of potential value to public health professionals, particularly in resource-limited contexts. The integration of both quantitative and qualitative data, along with an emphasis on real-world application, is commendable. However, a few remaining issues must be addressed before final acceptance.

1. Clarify and streamline the abstract

• The abstract is informative but could benefit from improved clarity and brevity. Certain terms such as “contrariwise” are less common in scientific writing and may hinder accessibility. Replace “contrariwise” with “alternatively” and consider condensing long sentences for improved readability.

*We have replaced “contrariwise” with “alternatively” and revised several long sentences to improve clarity and conciseness.*

***Lines 45-46, highlighted in yellow in the manuscript***

*“… and reluctance among families with middle-high socioeconomic status. Alternatively, opportunities included positive community perceptions …”.*

2. Refine discussion of results

• The discussion section contains repetition of results. While referencing findings is useful, repeating detailed data limits space for deeper interpretation and policy relevance.

• Instead of repeating knowledge percentages from the Results, use this space to propose how these gaps should inform health education initiatives.

*We have removed all repeated mentions of the results and instead added a deeper interpretation and discussion of the policy relevance of our findings. Additionally, we have created a dedicated Public Health Implications section to propose recommendations based on our study results (see below).*

3. Deepen policy implications in the conclusion

• The conclusion provides practical recommendations, but could be more action-oriented. A well-articulated roadmap strengthens the utility of the findings for policymakers and health system planners.

• Suggest launching a pilot community surveillance initiative integrating digital tools, with evaluation benchmarks based on this study.

*We have now recommended actionable strategies based on our findings to strengthen maternal and child health surveillance and service delivery, including the pilot community-based surveillance initiative suggested by the editor. These recommendations are compiled in a dedicated 'Public Health Implications' section at the end of the Discussion.*

***Line 449, highlighted in yellow in the manuscript***

***Public Health Implications***

*This study highlights several actionable strategies to strengthen maternal and child health surveillance and service delivery. First, targeted maternal and child health education, particularly for men on obstetric danger signs, alongside efforts to foster paternal involvement, should be promoted, as husbands play a crucial role in household decision-making and access to healthcare services. Second, to prevent under-surveillance problems among migrant populations, gated communities, and stigmatized cases, tailored approaches could work, including: (1) proactive outreach in densely populated rental housing clusters and informal settlements to better reach migrant households; (2) cooperation with resident associations and private clinics to improve surveillance participation among higher socioeconomic groups in gated communities; and (3) public campaigns to reduce stigma and normalize health-seeking behaviour to better to capture sensitive cases, e.g., unwanted pregnancies. Third, leveraging user-friendly digital tools such as mobile-phone-based data reporting systems may help increase participation in community-based surveillance, including in the three aforementioned groups.* *A pilot initiative integrating digital tools into community-based surveillance is recommended as the next step, with this study serving as a benchmark for future evaluations. Lastly, strengthening the role, capacity, and support for health volunteers is essential, and this can be achieved by providing regular training and basic equipment, genuine recognition, and small incentives (transport allowances) to sustain volunteer performance. These strategies can be implemented through intersectoral collaboration across the health, social welfare, education, and civil registration sectors.*

4. Enhance transparency of qualitative insights

• Although direct quotes have been added, representation of voices could be more balanced. Quotes are largely from officials or volunteers, with limited insights from family/community members. Consider incorporating more quotes from mothers or household heads to reflect end-user perspectives.

*We had already included community members in our qualitative analysis, as shown in* ***Table 3*** *and* ***Supplementary Table 1****. All three community member representatives interviewed were women, and most of them (2 out of 3) had lived in the neighborhoods for more than 10 years. We considered length of residence in our selection due to the city’s high migration rates, which may limit respondents’ familiarity with ongoing community issues.*

*Community insights were further strengthened by including* ***heads of neighborhoods or hamlets****, who provided broader community perspectives (also shown in* ***Table 3*** *and* ***Supplementary Table 1****). These individuals were appointed by the community to lead neighborhood activities. As they are not paid by the government or receive any formal remuneration, their neutrality is expected.*

*Nevertheless, we have added a suggestion for future studies to directly interview pregnant women, new mothers, and men or husbands as heads of households to better capture the end-user perspective, as in our study, data from these groups were collected only through a quantitative survey.*

***Lines 442, highlighted in yellow in the manuscript***

*“Although the quantitative survey included community members (heads of households) involved in community-based surveillance, the qualitative analysis has yet to incorporate perspectives from pregnant women, mothers with infants, or men as husbands. Future studies are encouraged to include these groups in qualitative interviews to better capture end-user perspectives.”*

5. Language and terminology refinement

• Some phrases may benefit from more precise or neutral terminology. Terms like “ignorance rates” (now corrected) and “exclusive neighborhoods” could be rephrased for neutrality and clarity. Use “less knowledgeable” instead of “ignorant,” and “gated communities” or “higher-income areas” instead of “exclusive neighborhoods.”

*We have replaced “exclusive neighborhoods” with “gated communities” in favor of a more objective term.*

***Lines 264, 389, 454, 458, and Table 3, highlighted in yellow in the manuscript***

*“There is a challenge in reaching the residents who live in gated communities, as they are generally very difficult to be visited.”*

*“Interestingly, we observed that households with higher socioeconomic status, or those residing in gated communities, were …”*

*“Second, to prevent under-surveillance problems among migrant populations, gated communities, and stigmatized cases, tailored approaches could work, …”*

*“… and private clinics to improve surveillance participation among higher socioeconomic groups in gated communities;”*

*“Residents with middle-high income socioeconomic status who live in gated communities are …”*

6. Reference formatting and integration

• Citations are appropriate, but integration in the narrative could be smoother. Over reliance on parenthetical citation disrupts narrative flow. • Use phrases such as “as shown by Sharma et al. (2023)” instead of placing multiple bracketed references at the sentence end.

• Supplemental tables provide valuable data but are not always clearly referenced in the main text. Cross-referencing enhances reader navigation and data transparency. • Add phrases like “(see Supplemental Table 2)” directly after discussing survey items.

*We have now integrated the citations into the narrative and ensured that the results presented in the main text are clearly referenced to the corresponding tables or supplementary materials.*

***Lines 357, 362, 371, 378, 385, 393, 399, 402, 409, 420, 427, highlighted in yellow in the manuscript***

*“…, our observation that men had less knowledge of warning signs in pregnancy, childbirth, and newborn care was consistent with a study from Mersha (2019), which reported that men were often less aware …”*

*“…, Rahman et al. (2018) found that pregnant women who were accompanied by their husbands …”*

*“Similarly, Kebede et al. (2022) also showed that low paternal involvement …”*

*“Rogeaux (2022) found that migrant children in urban areas had lower weight gain, ... Sharma (2023) attributed this to financial hardship, inadequate feeding practices, … Khan (2020) further noted that ‘urban poor’ populations with unfavorable social determinants of health (SDoH) are …”*

*“…, Kim (2019) reported that stigma towards unmarried mothers caused them to hide their pregnancy and avoided antenatal care.  Mukaliburt (2022) similarly observed that stigmatization toward …”*

*“Czeisler (2020) noted that approximately 12% of routine medical care, …, were delayed during the pandemic”*

*“For example, Roberts et al. (2020) and Jang (2019) reported that in the United States, survey response rates were particularly low among ... Van Loon (2003) highlighted that such non-response can bias prevalence estimates, …”*

*“Diese (2018) observed that mobile phones were widely perceived by the community as …. “*

*“Verity et al. (2022) emphasized that timely detection of disease through surveillance can lead to …”*

*“In Thailand, for example, Kaweenuttayanon et al. (2021) reported that during the COVID-19 pandemic, …”*

*“These successes were possible, as noted by Sakeah et al. (2021), largely driven by the health volunteers' ability to …”*

*“Nontapet (2022) and Owusu (2023) reported that community health workers perceived regular training as ...”*

*“Yet, as noted by Bezbaruah (2021), such training is not consistently provided.”*

*“Sunguya (2017) and Bezbaruah (2021) also highlighted that the lack of essential equipment, …”*

*“As also suggested by Sunguya (2017), Nontapet (2022), and Sakeah (2021), if the budget allows, small monetary incentives …”*

***Lines 165, 182, 192, 200, 208, 211, 215, 219, 222, 232, 268, highlighted in yellow in the manuscript***

*“The characteristics of the respondents for the in-depth interviews and FGDs were described in* ***Supplemental Table 1****.”*

*“As shown in* ***Table 1****, the quantitative study population has …”*

*“… was disproportionately higher in men (34.2%) than women (10.7%) (see* ***Table 2****).”*

*“…, and this is more so in male (23.2%) than female (7.0%) respondents (see* ***Table 2****).”*

*“…, and 59.9% disagree if childbirth is assisted by non-medical professionals (e.g., shamans) (see* ***Supplemental Table 2****).”*

*“…, the majority of (98%) respondents said that they would approve such monitoring to be conducted (see* ***Supplemental Table 3****).”*

*“The majority (>82%) also did not have problems obtaining healthcare services (see* ***Supplemental Table 4****).”*

*“…, health professionals (11.6-12.9%), and community leaders (11.0%-14.1%) (see* ***Supplemental Table 5****).”*

*“Qualitative Analysis: Barriers to Establishing a Community-Based Surveillance System for Maternal and Child Health* ***[Table 3]”***

*“…, as illustrated in the following direct quotes. Complete statements are available in* ***Table 3****.”*

Final Comments: Your manuscript has undergone meaningful improvement in response to previous reviewer suggestions. The addition of statistical significance tests, a dedicated conclusion, and clearer methodological explanations have substantially improved the manuscript’s quality. With **minor revisions** primarily around clarity, structure, and terminology, the manuscript will be well-positioned for publication. We appreciate your contribution to advancing maternal and child health systems, especially in underserved settings.

*We thank the Academic Editor for their overall support of our manuscript.*

**Reviewer's Comments and Responses to Questions*:***

1. If the authors have adequately addressed your comments raised in a previous round of review and you feel that this manuscript is now acceptable for publication, you may indicate that here to bypass the “Comments to the Author” section, enter your conflict of interest statement in the “Confidential to Editor” section, and submit your "Accept" recommendation.

Reviewer #1: (No Response)

Reviewer #2: All comments have been addressed

2. Is the manuscript technically sound, and do the data support the conclusions? The manuscript must describe a technically sound piece of scientific research with data that supports the conclusions. Experiments must have been conducted rigorously, with appropriate controls, replication, and sample sizes. The conclusions must be drawn appropriately based on the data presented.

Reviewer #1: Yes

Reviewer #2: Yes

3. Has the statistical analysis been performed appropriately and rigorously?

Reviewer #1: Yes

Reviewer #2: Yes

4. Have the authors made all data underlying the findings in their manuscript fully available? The PLOS Data policy requires authors to make all data underlying the findings described in their manuscript fully available without restriction, with rare exception (please refer to the Data Availability Statement in the manuscript PDF file). The data should be provided as part of the manuscript or its supporting information, or deposited to a public repository. For example, in addition to summary statistics, the data points behind means, medians and variance measures should be available. If there are restrictions on publicly sharing data—e.g. participant privacy or use of data from a third party—those must be specified.

Reviewer #1: Yes

Reviewer #2: Yes

5. Is the manuscript presented in an intelligible fashion and written in standard English? PLOS ONE does not copyedit accepted manuscripts, so the language in submitted articles must be clear, correct, and unambiguous. Any typographical or grammatical errors should be corrected at revision, so please note any specific errors here.

Reviewer #1: Yes

Reviewer #2: Yes

6. Review Comments to the Author

Please use the space provided to explain your answers to the questions above. You may also include additional comments for the author, including concerns about dual publication, research ethics, or publication ethics. (Please upload your review as an attachment if it exceeds 20,000 characters)

Reviewer #1: The authors have satisfactory addressed most of my previous recommendations, but I am not still convinced the flow of the discussion part. You don't have to mention study aim again in here to start with, at the discussion just mention your main findings, first finding and you interpretation flowed by comparing other studies. I would suggest to flow a clear line of presentation:

1.Your study results, two to three main findings 2. You can compare other studies to each point or as you did now mention all findings and then comparing the other studies to the equivalent finding.

3. Mention your limitations here. 4. Make your recommendations here or in the conclusion part at last section, not in the middle of the discussion as you mentioned in L366.

*We thank Reviewer #1 for their thorough and constructive feedback on our manuscript. In response, we have revised the Discussion section to improve its logical flow and coherence. Following the reviewer’s advice, we now begin the section by stating our main findings and have omitted the previously included study aim. This is followed by comparisons with other studies, a discussion of the study’s limitations, and a dedicated Public Health Implications section presenting our recommendations at the end of the manuscript.*

Reviewer #2: (No Response)

7. PLOS authors have the option to publish the peer review history of their article (what does this mean?). If published, this will include your full peer review and any attached files. If you choose “no”, your identity will remain anonymous but your review may still be made public. **Do you want your identity to be public for this peer review?** For information about this choice, including consent withdrawal, please see our Privacy Policy.

Reviewer #1: **Yes:** Abdulkadir Ismael Ahmed

Reviewer #2: No
